# Supplementary material for: The external realities of people with type 2 diabetes—Understanding disease perspective and self-management behaviour via Grounded Theory Approach
Source: PLoS One. 2021 Jan 14;16(1):e0245041. doi: 10.1371/journal.pone.0245041 (PMC7808602; doi:10.1371/journal.pone.0245041)
Supplement: S2 Table — (DOCX) [file pone.0245041.s004.docx]

**S2 Table : Selected quotations from subcategories related to the themes within the external environment**

| **Category** | **Sub-Category** | **Quotations** |
| --- | --- | --- |
| **Fixed background status**  Non-modifiable factors that shapes the daily lives of the patient | Lifestyle  Sociodemographics  Food Environment | At night sometimes something comes up, and I’ll have to go out. When I go out, I forget my medication  Sometimes at night we forget. We are too tired, we just go and sleep and forget to take medicine  When you sit down with the dietician and they tell you stuff, it’s good. But is it practical in real life or not? Unless you’re admitted in the hospital, right?  Working as a security guard, or having a stable 9 to 5 job, it’ll be easier to take medicine on time and regularly. But I’m a truck driver. It’s difficult  Back then, I was younger, I was ignorant (about the disease).  More educated people sometimes may even reject what the doctor says because they feel they know better  During the fasting month, I don’t take my medication…  I buy those glucometer strips, but they really are expensive  Sometimes when you come home from work, you don’t feel like doing exercise, especially when you come home late. You ask yourself ‘Why must I go out and do exercise?’, I feel my household chores are already a hand full  The food we buy is not healthy at all, but I have no choice  When you are outside, the temptation to taste the food is so strong  The government should impose heavy taxes on sugar. That would dissuade shops from making everything sweet  My wife says she will help prepare a much healthier diet while I am at home. So we ensure we take food which contains more fibre and lesser amounts of sugar. I avoid sugary beverages if I can. I try my levels best not to consume when I am out of the house because it’s a healthier eating at home. We eat more vegetables |
| **Personal experience**  Physical and emotional experience pertinent to the phenomenon of living with diabetes | Experiential  Adverse  Exemplar  Uncertainty  Wellbeing  Influence  Relationship with GPs  Service | I’m supposed to take two (tablets), but sometimes I reduce to one. But if I take two, I feel as if I cannot get up in the morning. I feel rather weak  Based on my experience, previous drugs didn’t really work for me. Only this current drug, I could see changes within the week  Maybe initially your body doesn’t quite accept the medication that we consume. But after 4 or 5 months or so, your body will eventually adapt to it  Back then, I could get things done fairly quick. But now, ever since I was diagnosed with diabetes, I dont’t appear to function like that anymore  When I recently started this new medication, I feel easily tired, and I become very sleepy  Then my eyesight had issues. The doctor told me it was because of my diabetes  You will start to feel it if you don’t take your medication. Lethargy, headaches and everything  I don’t really have any issues only that it hurts sometimes, when I inject (insulin) myself  Two of my brothers are gone (as a complication of diabetes). This makes me be more serious about my diabetes  I have a relative who’s been on insulin since the age of 8. He has to take insulin 4 times a day. And he has to restrict his diet and everything.  I’ve spoken to some friends. They have diabetes, and they control it really well. So even at an old age, they have really good control. That really motivates me.  I have heard people saying taking too much tablets can kill you at the end of the day.  My family members, especially my sister, she keeps telling me to avoid insulin and get a second opinion instead. When she knows I am going to meet a doctor, she insists that I tell them I am not willing to use insulin.  For now, I can still go about my activities. Tomorrow or the day after, I don’t know..  I keep asking myself, why is my blood sugar levels always high? I keep taking my medicine, yet it is always high  I keep asking myself, why is it always like that! Shouldn’t my blood sugar have gone down by now?  I know it is important to control my disease. I just feel afraid of the disease sometimes. I am just troubled by the fact that anything [complications] can occur at anytime [beyond control]  After I started doing it (insulin) properly, I saw some (positive) changes  When I didn’t change the needle frequently, it hurts. Now that I change the needle each time, I no longer feel any pain whatsoever  I feel much better right now [after taking medications]. I feel physically healthy and my work performance is much better. I no longer feel lethargic  I have not been compliant with my medications you know and my blood glucose readings [glucometer] were at 9 or 10. But then once I consume the blended bitter gourd juice, I realize the readings tend to normalize  I’ve heard many people tell me not to take all these medications. Don’t take it because end of the day, all these medicine have negative effects on your health  My sister told me to tell the doctor to not to provide me insulin if recommended  Sometimes, when I experience side effects, I feel like giving up. But my mom, she’s a nurse. She’s the one who motivates me to take my medication  I’d prefer if the doctor advises me. I’ll listen and follow  It all comes down to the relationship between doctor and patient. That determines the trust and everything else.  I dislike those kind of doctors. Doctors that say you have to do this otherwise you succumb to your disease. They force us to do something we don’t want to. The patient has rights, too!  The services provided here are excellent. I have no issues at all  I have no doubts about the quality of the medication given to us. The government is the one providing it, and they also want to do the best for us  So seeing a government doctor is like that. They look at you, look at your results, and prescribe that same medication for the next four months. But I don’t blame them, they have no time |
| **World view**  Self and society’s point of view about disease | Health Belief  Knowledge  Perception | If it’s vegetables and spices, I don’t mind taking, I know that certain things can help control sugar levels. But if it’s those traditional medicines, I don’t trust those.  Heart problems, cholesterol. These two are linked with uncontrolled diabetes.  I’ve heard many people say that taking too many medicines cause ill health at the end of the day  People consider us as having severely uncontrolled diabetes when we mention that we have started taking insulin  It’s the perception people have you know. Once you start taking insulin, they think that our disease is out of control and that anything can happen to us. It’s just a matter of time  I think even if you stopped taking any medicine, I am certain you can bring down your sugar levels - you just need to do exercise regularly and have restraint over your food intake. If you are consistent, I believe you can bring down your sugar levels.  I sweep, cook, and do the laundry everyday. I mean when I walk upstairs and down, I feel that is a form of exercise. I sweep the floor and clean the house. And I sweat it out too you know. I don’t know it’s true or not, but I think that’s enough for me  I know that this disease is hereditary  One of the complications is that your eyesight will get worse  From what I understand diabetes is this disease where our pancreas cannot produce insulin  I know diabetes runs in the family. If your dad has it, your mom has it, then 95% of the time you end up getting it too  I have this fear that diabetes will lead to kidney problems in the future. It will severely limit my daily activities as I might to undergo hemodialysis when that happens  When I look at myself, I realize I have poor control. Sometimes I consume my medications, sometimes I don’t  Yes. Common. Diabetes is very common in our society |
