# Supplementary material for: The external realities of people with type 2 diabetes—Understanding disease perspective and self-management behaviour via Grounded Theory Approach
Source: PLoS One. 2021 Jan 14;16(1):e0245041. doi: 10.1371/journal.pone.0245041 (PMC7808602; doi:10.1371/journal.pone.0245041)
Supplement: S3 Table — (DOCX) [file pone.0245041.s005.docx]

**S3 Table: Themes, Sub-Themes, Codes, Quotations within the Conceptual model (via Grounded Theory Approach)**

| **Core Phenomenon** | **Axial Coding** | **Open Coding (using gerunds)** | **Quotations** |
| --- | --- | --- | --- |
|  |  |  |  |
| Fixed background status  *Non-modifiable factors that shapes the daily lives of the patient* | Food Environment | Needing to eat out | “Sometimes we have our lunch outside,” – **Male, (IDM 002)**  “I normally eat outside.” – **Male (IDM 009)**  “We go outside and buy food, like lunch, for example.” – **Female (IDM 003)** |
|  |  | Feeling urge to violate diet when eating out | “When I’m out and I see the food, I want to eat it!” – **Male (IDM 002)**  “(When I’m outside) my wife will tell me take only one or two, but sometimes I indulge.” – **Male (IDM 014)**  “When you are outside, the temptation to taste the food is so strong, doctor.” – **Male (IDM 017** |
|  |  | Commenting about poor food environment (eating out). | “When we eat at the stalls outside, for breakfast, the food is all carbo.” – **Male (IDM 012)**  “The outside food is not healthy at all, but I have no choice. And when I see, I want to eat.” – **Male (IDM 002)**  “The problem with Malaysia is the food. It makes people sick.” – **Male (IDM 001)** |
|  |  | Blaming food vendors for adding too much sugar in food | “Sometimes they put more sugar in the drinks – **Male (IDM 002)**  “Sometimes they add sugar to the carbo!” – **Male (IDM 012)**  “When I’m out I see the vendors put 3 or 4 spoons of Milo into my Milo Kosong.” – **Male (IDM 017)** |
|  |  | Having positive home food environment helps | “When I’m at home my wife helps me control my diet.” – **Male (IDM 017)**  “I like a good variety of food. But not outside food. Healthy home cooked food.” – **Female (IDM**  “I can eat healthier food at home, and even encourage my son to eat healthy.” – **Male (IDM 014)** |
|  |  | Needing proper legislation to monitor food environment | “Perhaps if the government can impose laws that state all food stalls close at 10 and open at 6, it would help.” – **Female (IDM 011)**  “The government should impose heavy taxes on sugar. That would dissuade shops from making everything sweet.” – **Male (IDM 015)** |
|  | Information | Obtaining Information (Internet) | “Yeah one of the places I get my info from is the internet.” – **Male (IDM 018)**  “Any information I need, I just Google.” – **Male (IDM 004)**  “Back when my eyes were okay, I used to Google and read about diabetes.” – **Female (IDM 006**) |
|  |  | Obtaining information about CAM (Internet) | “I’ve seen on Whatsapp and Facebook, various alternatives, herbal remedies to cure diabetes.” – **Female (IDM 005)**  “There’s a lot of ads on Facebook. All these herbs and whatnot.” – **Female (IDM 022)**  “Traditional medicine from India, a lot of it comes on the internet, Whatsapp and all.” – **Male (IDM 013)** |
|  |  | Gaining information about CAM (friends) | “My friends gave me the idea of cutting up Okra and drinking it to reduce blood sugar.” – **Male (IDM 012)**  “Many people have recommended traditional medicine to me…” – **Female (IDM 006)**  “Even though people have introduced alternative medicine saying it’ll reduce my blood sugar, I’ve never touched it.” – **Female (IDM 005)** |
|  |  | Obtaining information from medical programs (TV) | “I also watch a lot of programs, medical programs on TV.” – **Female (IDM 011)**  “I get all this information from the television and other sources” – **Male (IDM 016**)  “There are specific shows on TV that talk about diabetes. I watch that.” – **Male (IDM 018)** |
|  |  | Obtaining information (Book) | “You know, sometimes books and booklets I read when I go to the clininc.” – **Male (IDM 010)**  “I read a lot of books as well and that’s where I get me information.” – **Female (IDM 011)**  “Then after reading books about diabetes, I realized what I had to do.” – **Male (IDM 023)** |
|  |  | Obtaining Information (Medical Pamphlets) | “Those catalogues (pamphlets) at the hospital? I read about the disease and how to control it…” – **Male (IDM 018)**  “Sometimes you go to the clinic you find those brochures about controlling your blood sugar…” – **Male (IDM 017)**  “Sometimes when I go to the pharmacy, I ask for those medication inserts, so that I can read.” – **Male (IDM 008)** |
|  |  | Obtaining information (Newspaper) | “Sometimes in the newspaper they have the health column, diabetes, blood pressure, it’s all written there.” – **Male (IDM 002)**  “… also by reading. Reading the newspapers and all that.” – **Male (IDM 016)**  “Sometimes the newspapers, I read about diabetes and the food to eat and all that.” – **Female (IDM 020)** |
|  |  | Obtaining information (Family) | “I receive information about certain complications, such as kidney and all these things, from friends and family members.” – **Male (IDM 010)**  “I sometimes get information from others… Others like family members and all.” – **Male (IDM 016)**  “I consult my brother lah. He’s very knowledgeable about medicine and all that.” – **Female (IDM 022)** |
|  |  | Obtaining information (HCP) | “When I go for my follow ups at the hospital, the doctors educate me.” – **Female (IDM 006)**  “I will ask the doctor, whether private or government. I will ask the doctor.” – **Male (IDM 023)**  “Yeah I usually get all my info from my doctor.” – **Female (IDM 019)** |
|  | Lifestyle | Missing medication when away from home | “At night sometimes something comes up, and I’ll have to go out. When I go out, I forget my medication.” – **Male (IDM 010)**  “I rarely miss my medication, unless I go out somewhere, like a wedding or a meeting.” – **Female (IDM 011)**  “The medication that we have to take, we’re bound to forget when we are out.” – **Male (IDM 023)** |
|  |  | Missing medications due to hectic lifestyles | “It’s not that I don’t want to take. But you tend to focus on other things, and neglect your own health.” – **Male (IDM 012)**  “Sure you’ll forget once or twice, perhaps when you’re busy.” – **Male (IDM 009)**  “Sometimes I’m so busy I forget.” – **Male (IDM 016)** |
|  |  | Missing medication prior to sleeping hours | “Sometimes at night we forget. We are too tired, we just go and sleep and forget to take medicine.” – **Female (IDM 003)**  “When I’m too sleepy, I forget to take my medicine and just go and sleep.” – **Female (IDM 022)**  “Sometimes I sleep at 8.30pm and wake up at 2am. How am I supposed to take medicine?” – **Female (IDM 024)** |
|  |  | Missing medication when tired | “I miss my medications sometimes because I’m just too tired.” – **Male (IDM 018)**  “After work you come back, you’re just too tired to take the rest of the medication you’re supposed to take.” – **Male (IDM 012)**  “Sometimes I’m just so tired, too tired to take medicine.” – **Female (IDM 005**) |
|  |  | Forgetting to bring medication along when out of home | “I try to put it in a bag and take it along when I go out, but sometimes I don’t.” – **Male (IDM 009)**  “…and then I’ll forget to take my medication along with me.” – **Female (IDM 005)**  “Sometimes you go outstation you forget to pack your medication along.” – **Male (IDM 016)** |
|  |  | Denying work activities lead to missing medication | “Ah, no, work doesn’t influence my medication because I make sure I take each day.” – **Male (IDM 008)**  “I don’t think work influences medication. It all comes down to the person.” – **Female (IDM 024)**  “No it doesn’t affect me much because I know where I want to go, at what time, and when to take my medication.” – **Female (IDM 020)** |
|  | Practicality | Experiencing lesser inconvenience with oral medication | “Pills are easy. Keep them in our pockets and swallow them.” – **Male (IDM 015)**  “Tablets are general medication. Anywhere can take. Insulin is a little more personal.” – **Female (IDM 020)**  “Tablet we just have to swallow. No issues.” – **Female (IDM 024)** |
|  |  | Taking medication in the morning is easy | “In the mornings I don’t have any problems (taking my medication). Morning, after breakfast I take my medication immediately.” – **Male (IDM 002)**  “Generally my medications in the mornings are okay lah.” – **Male (IDM 014)**  “Morning sure take. Night only lah (got problem)” – **Female (IDM 003)** |
|  |  | Rejecting the practicality of dietary advice | “When you sit down with the dietician and they tell you stuff, it’s good. But is it practical in real life or not? Unless you’re admitted in the hospital, it isn’t.” – **Male (IDM 012)**  “If I eat the diet that is suggested, I will not be full. I won’t have energy at work.” – **Male (IDM 015)**  “If I follow the dietary advice given to me 100%, one day I’ll starve!” – **Male (IDM 009)** |
|  |  | Maintaining exercise as a habit is difficult | “Not every day we can exercise. Because we got family, we got other commitments…” – **Male (IDM 013)**  “I’m lazy sometimes (to exercise). – **Female (IDM 005)**  “Practically, I don’t do exercise everyday.” – **Female (IDM 011)** |
|  |  | Blaming work constraints for poor dietary adherence | “I work till about 8. I need to eat. If a car doesn’t have fuel it cannot run. How am I to work if I don’t eat?” – **Male (IDM 015)**  “I’m a truck driver. I travel from Butterworth to Singapore. I have to eat.” – **Male (IDM 009)**  “…in a different environment, the person cannot have all his meals planned out. He has to work.” – **Male (IDM 012)** |
|  |  | Feeling inconvenience injecting insulin on the go | “Insulin you have to bring it here, bring it there. Feel a little sick of it.” – **Male (IDM 018)**  “Its really inconvenient to inject myself in the car when I get posted out.” – **Male (IDM 004)**  “When I’m out attending meetings and things like that, it’s difficult to take insulin.” – **Female (IDM 021)** |
|  |  | Having stable work routine affects medication compliance | “When I’m more organized, it’s easier la (taking medication). If I have to suddenly do things, then it’s difficult.” – **Female (IDM 021)**  “Working as a security guard, or having a stable 9 to 5 job, it’ll be easier to take medicine on time and regularly. But I’m a truck driver. It’s difficult.” – **Male (IDM 007)**  “When I’m busy it’s hard. I forget, or I’m tired. Now that I’m at home, it’s easy to take my meds. My meds are always beside me, its easy to follow timing.” – **Female (IDM 019)** |
|  | Relationship | Thinking role of doctor as advisor | “The doctor will be the one to say ‘okay, this is your condition.’” – **Female (IDM 021)**  “I prefer it if the doctor advises me. I’ll listen and follow.” – **Male (IDM 023)**  “The doctor gives fatherly advice. So we have to follow.” – **Male (IDM 016)** |
|  |  | Building rapport with doctor has positive effect | “If you force the person to take medication, they will not want to take it. If you tell them nicely, chances are they’ll want to cooperate.” – **Male (IDM 012)**  “It all comes down to how the relationship between doctor and patient is. That determines the trust and all.” – **Female (IDM 003)**  “Having a good rapport with the doctor will make us more confident in asking questions, and it will affect how we take our medication (in a positive manner).” – **Female (006)** |
|  |  | Being courteous has positive effect on patients | “If the doctor is kind with his words, we will be more receptive.” – **Male (IDM 012)**  “If the doctor is nice, it will improve the control as well.” – **Female (IDM 003)**  “If the doctor speaks to us nicely, we also won’t be stressed.” – **Male (IDM 023)** |
|  |  | Being rude or disparaging to patients during consultation | “There are doctors that threaten patients…” – **Female (IDM 003)**  “I’ll speak frankly, doctor. There are some doctors that are so rude!” – **Female (IDM 011)**  “The doctor told me that she hoped I wouldn’t come back in a wheel chair. I was so disappointed with the way she spoke.” – **Male (IDM 015)** |
|  |  | Feeling upset with the doctor | “Doctor told me I will die. I didn’t reply but I was really heartbroken. I started to cry.” – **Female (IDM 006)**  “The doctor asked me if I wanted to die. That, I feel, is a very bad thing to ask me.” – **Male (IDM 023)**  “There are a lot of things that the doctor said when he scolded my mom. I got very pissed off.” – **Female (IDM 005)** |
|  |  | Disliking condescending attitude of doctors | “I dislike those kind of doctors. Doctors that say you have to do this otherwise you die. They force us to do something we don’t want to. The patient has rights, too!” – **Female (IDM 003)**  “Even here there are doctors that tell us patients that if we don’t take the medicine, we will die very quickly.” – **Female (IDM 006)**  “I don’t like it doctor, when the other doctors treat me like that. They say if I want to die, no need to take their medication.” – **Female (IDM 022)** |
|  |  | Feeling doctors behavior can be unpredictable | “Some doctors are nice. They go the extra mile. But some doctors are very cold.” – **Male (IDM 008)**  “Some doctors are very strict. Some are very nice. Some accuse us.” – **Female (IDM 022)**  “They way they communicate is very different depending on doctors.” – **Female (IDM 005)** |
|  |  | Having poor trust (doctor) | “Some doctors say one thing, some say another. So I don’t know if these doctors are telling the patients the real truth or not.” – **Male (IDM 010)**  “This one time I had a fracture, they told me it was a tear. So, like, doctors these days don’t really have a lot of skill. Can’t even read an x ray.” – **Male (IDM 015)**  “This one doctor, he was very young lah. He told me that my wife’s leg had to be amputated. But then I went private and he cured her leg.” – **Male (IDM 023)** |
|  |  | Receiving reprimand from doctor (patient deserving) | “I’ll be honest with the doctor (about my control). Of course I’ll get shelling (scolding), but that’s part of the relationship.” – **Male (IDM 012)**  “The doctor scolds me because of my poor control. But she’s doing it for my own good. So I’m trying to follow.” – **Male (IDM 009)**  “When the doctor scolds me, I cannot take offence. I work as well, I too scold people who don’t listen to my advice.” – **Female (IDM 024)** |
|  |  | Being receptive to opinions from other T2D patients | “Yeah, when we discuss our health issues with other sick people, it’ll give us more knowledge.” – **Male (IDM 002)**  “The more people tell me about their diabetic condition, the more I understand…” – **Female (IDM 021)**  “It’s very helpful to have friends who have the same disease, because we can listen to what they say and make our own conclusions.” – **Male (IDM 016)** |
|  |  | Exchanging information openly with other T2D patients | “I told him the effect of diabetes on his various organs, and all the complications that can happen. Shock therapy.” – **Female (IDM 020)**  “Some of my colleagues hide the fact that they are diabetic. I told them that they are wrong. You should share the fact openly.” – **Male (IDM 023)**  “Us friends who have diabetes, we sit together and exchange views on the disease.” – **Male (IDM 002)** |
|  | Service | Praising services at most clinics (Satisfied) | “The services provided here are excellent. I have no issues with it.” – **Male (IDM 017)**  “I will say it (the service provided) is good. 100%. Everyone really cares and helps the patient recover.” – **Male (IDM 013)**  “Good. Here they will give you a good check up, and do what is necessary.” – **Male (IDM 001)** |
|  |  | Praising the quality of medication given at government clinics | “I feel that the medication from this clinic is really good.” – **Male (IDM 015)**  “Yeah the medication is good lah.” – **Male (IDM 007)**  “I have no doubts about the quality of the medication given to us. The government is the one providing it, and they also want to do the best for us.” – **Male (IDM 016)** |
|  |  | Seeking treatment at private clinics | “When you go to a private clinic, they give you the red carpet treatment.” – **Male (IDM 014)**  “Sometimes I go to a private doctor…” – **Female (IDM 003)**  “Took my wife to the private doctor, I saw how he took care of my wife, cleaned her leg up. Within a few weeks, her recovery was noticeable. No need for amputation.” – **Male (IDM 023)** |
|  |  | Hurrying consultation with patients | “So seeing a government doctor is like that. They look at you, look at your results, and prescribe that same medication for the next four months. But I don’t blame them, they have no time.” – **Male (IDM 010)**  “I don’t have much of a relationship with my doctors. They just look, prescribe the medication, and I go. Some of them don’t even talk to you.” – **Female (IDM 003)**  “When I’m here I don’t talk much with the doctors. They just tell me what I need to do, and that’s it. Finished.” – **Female (IDM 005)** |
|  |  | Receiving inadequate care because of handling too many patients | “Probably because they are under tension of seeing too many patients. When you see the doctor, he just tells you how you are, gives you medication and you go. That’s all.” – **Male (IDM 002)**  “If I ask too many questions, the doctor says he’s got no time.” – **Male (IDM 010)**  “Maybe he (doctor) is too busy with many patients, he doesn’t have the time to explain anything to me.” – **Female (IDM 011)** |
|  |  | Seeing different doctors during each consultation | “Difficult to form relationships because the doctors change each time.” – **Female (IDM 020)**  “The doctors keep changing, so I can’t really comment…” – **Female (IDM 005)**  “Each time I come, I don’t get the same doctor…” – **Female (IDM 011)** |
|  |  | Confusing because receive different opinions from different doctors | “My body can’t handle it. This doctor has one opinion, another doctor will have another opinion.” – **Female (IDM 019)**  “One doctor in KL told me about my surgery, different from what the doctors at the hospital told me.” – **Male (IDM 013)**  “Some doctors recommend different medication from other doctors…” – **Female (IDM 003)** |
|  |  | Seeking alternative opinions from other doctors | “Its always good to get a second opinion. Then we can compare…” – **Male (IDM 013)**  “I prefer seeing more than one doctor. I get a different opinion about which medication is better and things like that.” – **Female (IDM 0030)**  “I you get a new doctor, then sometimes the new guy can find out what the other guy missed.” – **Male (IDM 014)** |
|  | Demographics | Ignoring consequence of disease when young | “Back then, I was younger, I was ignorant (about the disease).” – **Female (IDM 006)**  “When we are younger we don’t really care…” – **Male (IDM 017)**  “Younger people skip their medication more often because they feel they are healthy.” – **Male (IDM 016)** |
|  |  | Believing age plays no role in T2D management | “I do not think age influences us.” – **Male (IDM 012)**  “The management doesn’t depend on age. It depends on how serious the disease.” – **Female (IDM 011)**  “I feel age does not limit us in any way. It’s all up to our thinking.” – **Male (IDM 015)** |
|  |  | Getting old makes you more concerned about health | “Maybe because we’re older, we’re more aware of our health…” – **Female (IDM 019)**  “But now that I’m nearing 40, I’m a lot more concerned about my health.” – **Female (IDM 024)**  “When you grow older, you become a lot more disciplined (about your disease management and health).” – **Male (IDM 017)** |
|  |  | Believing education plays no role in T2D management | “You don’t need an educated background (to manage your disease properly).” – **Male (IDM 001)**  “I don’t think that education plays a part, doctor. I believe it’s all up to the individual.” – **Male (IDM 017)**  “Doesn’t matter. Highly educated, or not educated, everyone has to follow what the doctor says.” – **Male (IDM 009)** |
|  |  | Making own decisions if higher level of education | “Those who are slightly smarter tend to do their own thing.” – **Female (IDM 021)**  “More educated people sometimes may even reject what the doctor says because they feel they know better.” – **Male (IDM 007)**  “If you’re highly educated, you may not accept some of the advise given to you…” – **Female (IDM 020)** |
|  |  | Following doctor’s advice better if have lower education | “Like my mother, people who have lower education tend to treat the doctor’s words as Gospel.” – **Male (IDM 012)**  “Like me lah. We study less, so we don’t know, we just do whatever the doctor advises.” – **Female (IDM 003)**  “Someone averagely educated will tend to follow the doctor’s advice.” – **Male (IDM 018)** |
|  |  | Exploring information if education level is higher | “People with higher levels of education tend to do their own research into the drugs they take.” – **Male (IDM 018)**  “The person who is more educated will probably find out what’s good for him and what’s not.” – **Male (IDM 016)**  “The smarter people are more aware of their disease.” – **Female (IDM 019)** |
|  |  | Believing patients with lower education have poor knowledge | “People with lower education don’t really understand what their disease is all about.” – **Male (IDM 012)**  “But he’s an averagely educated man lah. He doesn’t know about insulin and the pancreas and anything.” – **Male (IDM 008)**  “You have to make a lot of changes to your lifestyle. If you’re not very well educated, you won’t think this way.” – **Female (IDM 011)** |
|  |  | Fasting influences insulin taking ambiguity | “During the fasting month, I don’t take my medication…” – **Male (IDM 004)**  “I’m supposed to take the jab before I eat. But when I’m fasting, I don’t eat, how to jab?” – **Male (IDM 014)**  “So to me, when I take insulin, I cannot fast…” -  **Female (IDM 022)** |
|  |  | Seeking treatments at public clinic due to cost | “Better just go this side la (government). Why go private and waste money? Private is very expensive.” – **Female (IDM 003)**  “I’m grateful for what the government does for senior citizens like me. We don’t have to pay a single cent. If you go private, it costs a bomb.” – **Male (IDM 008)**  “That’s the good thing about the government. It is very cheap. Pay RM 1 only.” – **Male (IDM 001)** |
|  |  | Having no choice but to take suboptimal medications due to cost | “So what to do, poor people must go to poor man’s hospital (government hospitals) and take whatever they give lah.” – **Male (IDM 010)**  “I feel that they used to give better medicine back then. Expensive medication. Nowadays we get cheaper meds.” – **Female (IDM 020)**  “When I compare with my neighbor, he goes private, he gets better medicine. It’s more expensive, but he is paying for it lah.” – **Male (IDM 008)** |
|  |  | Lamenting over the cost of glucometer strips | “Those strips really are expensive.” – **Male (IDM 004)**  “I buy those strips, but they really are expensive.” – **Female (IDM 019)**  “Oh yes, those strips are expensive. They cost a small fortune.” – **Male (IDM 008)** |
| Personal Experience  *Physical and emotional experience pertinent to the phenomenon of living with diabetes* | Experiential | Having difficulty adhering to medication timing | “I do take care. It’s just that sometimes I don’t adhere to the proper timing…” – **Male (IDM 023)**  “I have a son with special needs. I need to take care of him and to care for all his needs. So because of that, it’s hard to follow the fixed time set by the doctor.” – **Male (IDM 016)**  “I forget to take my medication on time.” – **Female (IDM 020)** |
|  |  | Experiencing no side effects taking medication | “I experience no side effects with my medicine.” – **Female (IDM 003)**  “Side effects? No, doctor, I don’t experience any.” – **Male (IDM 015)**  “So far, I’ve been taking the hospital medicine, no side effects.” – **Male (IDM 009)** |
|  |  | Reducing medication dose due to side effects | “I’m supposed to take two (tablets), but sometimes I reduce to one. If I take two, I feel as if I cannot get up. Some sort of weakness…” – **Female (IDM 011)**  “I reduce my medication dose so that I don’t experience hypo at night.” – **Male (IDM 023)**  “Doctor asked me to take two tablets. But if I take two, I have severe dizziness. So I take one instead.” -  **Male (IDM 007)** |
|  |  | Knowing subjective response of medication on self | “I don’t know about others, but when I eat Metformin, my weight goes up.” – **Male (IDM 013)**  “Based on my experience, previous drugs didn’t really work for me. Only this current drug, I could see changes within the week.” – **Male (IDM 004)**  “It may vary from person to person. Some may react differently. For me, I know, 2 tablets is the best for my body.” – **Male (IDM 012)** |
|  |  | Taking time for medication to work | “…then I took my medication. It took a while, but eventually I started becoming healthy.” – **Male (IDM 007)**  “Maybe initially you don’t agree with the medication. After 4 or 5 months, your body will agree to it.” – **Female (IDM 011)**  “You need to be disciplined and take it. Only after a few weeks you’ll see change.” – **Male (IDM 012)** |
|  |  | Realizing elevation in blood glucose when skip medication | “I have the strip, so I test myself. If I don’t take my medication, the readings are high.” – **Male (IDM 023)**  “So I’ve tested it out. When I don’t take my medications, and I go to the clinic, the readings are very high.” – **Male (IDM 015)**  “Oh definitely. If I don’t take my medications for two days, my blood sugar will start to be high.” – **Female (IDM 011)** |
|  |  | Attributing inability to work to T2D | “When I do my job, especially a strenuous job, compared to a normal person, I get tired a lot faster.” – **Male (IDM 012)**  “Back then I can quickly get things done. But now, ever since I got diabetes, I can’t work like that anymore.” – **Male (IDM 007)**  “Without diabetes, you feel very active. With diabetes, you cannot do difficult jobs.” – **Male (IDM 009)** |
|  | Influence | Receiving incorrect advice from T2D patients | “My friend told me that all these medication will clog up my blood. And he advised me to not take medication according to the prescribed dose.” – **Male (IDM 015)**  “I’ve heard many people tell me not to take all these medications. Don’t take it because end of the day, all these medicine will kill you.” – **Female (IDM 005)**  “My friends all tell me not to take too many tablets, because it will spoil your kidneys.” – **Male (IDM 016)** |
|  |  | Being discouraged by others from taking insulin. | “…he disagrees with insulin. He doesn’t take.” – **Female (IDM 003)**  “My sister told me to tell the doctor not to give me insulin, like that.” – **Female (IDM 020)**  “So my friend told me not to take insulin anymore.” – **Female (IDM 024)** |
|  |  | Influencing medication taking habits | “Even my son occasionally checks up on me and asks if I have been taking my medication.” – **Male (IDM 004)**  “I have friends, from work. People whose sugar is 20 something. My sugar is around 9 – 11. So that motivates me to take my medicine.” – **Male (IDM 017)**  “Sometimes, when I experience side effects, I feel like giving up. But my mom, she’s a nurse. She’s the one who motivates me to take my medication.” – **Female (IDM 021)** |
|  | Adverse | Experiencing side effects | “When I recently started this new medication, I feel easily tired, and I become very sleepy.” – **Male (IDM 004)**  “Sometimes, when I take metformin on an empty stomach, I feel nauseous, doc.” – **Male (IDM 008)**  “I’m taking the jab now, and I get hypo very often. Almost every morning.” – **Male (IDM 014)** |
|  |  | Having complications (eye, neuropathy, wound healing) | “Then my eyesight had issues. The doctor told me it was because of my diabetes.” – **Female (IDM 006)**  “My skin had wounds that won’t heal…” – **Male (IDM 007)**  “The numbness really bothers me, doctor. My hands feel numb.” – **Female (IDM 022)** |
|  |  | Feeling pain injecting insulin into abdomen | “I don’t really have any issues only that it hurts sometimes, when I inject myself.” – **Male (IDM 023)**  “Yes, insulin is painful. I’ve even bled before.” – **Female (IDM 006)**  “That insulin, it hurts, doctor.” – **Female (IDM 024)** |
|  |  | Experiencing physical symptoms after missing medication for extended period of time | “You will start to feel it if you don’t take your medication. Lethargy, headaches and everything.” – **Female (IDM 021)**  “If I don’t take it for a day or two, I’ll feel it. Dehydrated, needing to urinate a lot…” – **Male (IDM 023)**  “If I don’t take medicine accordingly, I’ll feel tired and weak.” – **Male (IDM 015)** |
|  |  | Experiencing physical symptoms with uncontrolled T2D | “Earlier when I had diabetes, no matter where I was I felt sick. Lethargic.” – **Male (IDM 007)**  “That time, when I had diabetes, I felt very giddy.” – **Male (IDM 002)**  “Yeah if my blood (sugar) gets to around 20, my body feels really different.” – **Male (IDM 004)** |
|  |  | Acknowledging T2D usually shows no physical symptoms | “Even when I was diagnosed with diabetes, I felt totally normal.” – **Male (IDM 001)**  “For diabetes, you won’t really anything till the disease is much worse.” – **Female (IDM 022)**  “Diabetes initially has no effects on your body.” – **Male (IDM 018)** |
|  |  | Determining blood glucose level based on physical symptoms | “… so when I feel uncomfortable, then I will take my meds.” – **Male (IDM 023)**  “I trust my own body rather than the glucometer.” – **Male (IDM 012)**  “Yeah, I believe so. When I feel something isn’t right, and then I check, true enough, my sugar is high.” – **Female (IDM 011)** |
|  |  | Experiencing no physical symptoms with suboptimal T2D control | “When I feel something about me is not alright, I will go and check my sugar.” – **Male (IDM 010)**  “When I experience certain symptoms, I get curious and go and check my blood” – **Female (IDM 011)**  “…we will experience certain things. Then I’ll go and check, and sure enough, the readings will be high.” - **Male (IDM 023)** |
|  |  | Experiencing hypoglycemia | “I didn’t eat, then took my insulin. I went hypo.” – **Female (IDM 019)**  “…yeah I know I had good control, because several times I went hypo.” – **Male (IDM 024)**  “Sometimes mix up the medication dose, or the timing. Then I go hypo.” – **Female (IDM 022)** |
|  |  | Experiencing physical symptoms | “I’m embarrassed, because my wife is still young, and yet I can’t have sex with her.” – **Male (IDM 007)**  “That time I first experienced it. I had no sensation on my hands.” – **Female (IDM 019)**  “At night every few hours I wake up to go to the toilet.” – **Male (IDM 023)** |
|  | Exemplar | Witnessing complications in others | “I go to the hospital and see, kidney problems due to diabetes, amputated feet due to diabetes…” – **Male (IDM 023)**  “He (father) went through kidney failure and dialysis.” – **Female (IDM 005)**  “I know the complications, because I’ve seen it happen in others.” – **Male (IDM 016)** |
|  |  | Witnessing death due to T2D | “I have 3 brothers who are diabetic. Two of them are gone.” – **Male (IDM 010)**  “My sister had good (diabetic) control, but she passed away after some time. She couldn’t cope.” – **Female (IDM 011)**  “I have a friend. He’s 25 years old, just passed away. Diabetes.” – **Female (IDM 006)** |
|  |  | Witnessing complications or death reinforces T2D control | “…two of my brothers are gone. This makes me be more serious about my diabetes.” – **Male (IDM 010)**  “After witnessing all these complications happening to others, I got scared.” – **Female (IDM 019)**  “My sister in law recently got a stroke. So I realized I have to take my medication” – **Male (IDM 017)** |
|  |  | Being a role model | “…I’ll sit him down, and share my experience as a diabetic patient with him.” – **Female (IDM 06)**  “I advised my friend and just be straightforward with him about the reality of the disease.” – **Female (IDM 024)**  “Like now, I advise my kids that they have to do prevention.” – **Male (IDM 008)** |
|  |  | Learning from experience of exemplary patients | “I have a relative who’s been on insulin since the age of 8. He has to take insulin 4 times a day. And he has to restrict his diet and everything. From such a young age he’s experiencing that…” – **Female (IDM 011)**  “I’ve spoken to some friends. They have diabetes, and they control it really well. So even at an old age, they have really good control. That really motivates me.” – **Female (IDM 006)**  “I know this one lady, she’s 80 years old. She has really good diabetes control. She only takes metformin, and she manages her diet really well. Even though she’s 80, she can still control very well.” – **Male (IDM 013)** |
|  |  | Having family members with T2D | “I have a brother with diabetes.” – **Female (IDM 024)**  “I’m afraid of this sickness because my mother has it.” – **Male (IDM 017)**  “And I experienced how my dad went through diabetes.” – **Female (IDM 005)** |
|  |  | Witnessing others injecting insulin before | “I used to see my mother injecting herself with insulin. I felt really sorry for her.” – **Male (IDM 004)**  “….My brother takes insulin, …” – **Male (IDM 010)**  “My brother also takes insulin, but he’s still suffering…” – **Female (IDM 003)** |
|  | Uncertainty | Feeling uncertain of the nature of disease | “…I don’t know what’s going to happen. I’ve seen cases where everything is normal, and the next minute they get a stroke.” – **Male (IDM 016)**  “What’s happening inside my body, I don’t know. Only the doctor knows…” – **Female (IDM 019)**  “For now I can still go about my activities. Tomorrow or the day after, I don’t know...” – **Female (IDM 006)** |
|  |  | Frustrating unable to control blood glucose levels | “Even till now it only goes down to about 8. No lower.” – **Male (IDM 018)**’  “I keep asking myself, why is it always like that! Shouldn’t my blood sugar have gone down?” – **Male (IDM 004)**  “I keep asking myself, why is the blood sugar always high? Take medicine also, always high.” – **Female (IDM 003)** |
|  |  | Failing to understand poor control despite medication compliance | “I can’t understand why, even with all the medication, we can’t seem to be normal.” – **Male (IDM 001)**  “They ask me to come back frequently for controls. But no matter what, my blood sugar never goes down.” – **Male (IDM 007)**  “Then the doctor increases my meds to the maximum. And I take them. But I still don’t feel good.” – **Female (IDM 021)** |
|  |  | Accepting that fate is beyond our control | “Everyone dies. It’s just how you die, whether because of diabetes or other causes lah.” – **Male (IDM 010)**  “There are some people who really take care, and yet it (diabetes) happens. Its just too bad.” – **Male (IDM 014)**  “If it’s time to go, it’s time to go.” – **Female (IDM 003)** |
|  |  | Feeling uncertain about the effectiveness of insulin | “My brother is taking insulin, but he’s still suffering.” – **Female (IDM 003)**  “Insulin… you don’t know if it’s really helping you…” – **Male (IDM 016)**  “I look at my brother in law. Whether he takes insulin or not, I don’t see any difference.” – **Male (IDM 022)** |
|  | Wellbeing | Seeing improvements when adhere to proper insulin technique | “After I started doing it (insulin) properly, I saw some changes.” – **Male (IDM 018)**  “After I did that (take insulin as advised) my body felt good. My blood sugar went down…” – **Male (IDM 007)**  “When I didn’t change the needle, it hurt. Now that I change the needle each time, it goes in smoothly, no pain.” – **Male (IDM 009)** |
|  |  | Experiencing positive effects using CAM | “I feel that the traditional medicine can really help control blood sugar. And it also makes the body feel good.” – **Male (IDM 004)**  “I take both hospital and traditional medicine. And I feel more energetic when I take both together.” – **Male (IDM 002)**  “I take ‘ketum’ and I feel it’s good. Traditional medicine is more naturally suited for the body.” – **Female (IDM 024)** |
| World View  *Self and society’s point of view about disease* | Perception | Perceiving self to have poor disease control | “When I look at myself, I realize I have poor control. Sometimes I eat my meds, sometimes I don’t…” – **Male (IDM 015)**  “No, I don’t control my sugar well.” – **Male (IDM 007)**  “So far my sugar control is… average. I can’t get it down.” – **Male (IDM 017)** |
|  |  | Perceiving self to have good disease control | “I think it’s okay, I think it’s (diabetes) under control.” – **Female (IDM 003)**  “I think it’s (diabetes) well managed.” – **Female (IDM 011)**  “After my appointment my doctor saw that my control was good.” – **Female (IDM 020)** |
|  |  | Having T2D is common or normal in the society | “I feel diabetic is a norm, because I ask and they have.” – **Male (IDM 014)**  “Yes. Common. Diabetes is very common in our society.” – **Female (IDM 011)**  “I too feel that it’s becoming a norm in our society.” – **Male (IDM 015)** |
|  |  | Devolving autonomy to doctors | “I don’t know anything about the medicine. When I come here, the doctor asks me to take it, I take it. I don’t look into it too much.” – **Male (IDM 010)**  “I don’t study medicine and all this. If the doctor asks me to take, I take it.” – **Female (IDM 003)**  “The doctors know what is good for you. You follow lah.” – **Male (IDM 016)** |
|  | Health Belief | Trusting CAM if origin is from natural sources | “If it’s vegetables and spices, I don’t mind taking, I know that certain things can help control sugar levels. But if it’s those traditional medicines, I don’t trust.” – **Female (IDM 011)**  “Yeah, like some of those vegetables that people say are good for diabetes, I eat more.” – **Female (IDM 006)**  “Certain spices, like cinnamon. It helps control sugar levels. I take it.” – **Female (IDM 020)** |
|  |  | Having positive attitude towards CAM | “I think traditional medicine is good also.” – **Male (IDM 009)**  “I believe in traditional medicine. Why not? Indian traditional medicine is the oldest in the world. And people are going back to it.” – **Female (IDM 020)**  “If you say only medicine works, I say that’s wrong. Sometimes we can take other herbs and remedies and it helps the body.” – **Male (IDM 013)** |
|  |  | Believing T2D is linked to other diseases | “…heart disease, and other diseases, all linked with diabetes.” – **Female (IDM 021)**  “Heart problems, cholesterol. These two are linked with uncontrolled diabetes.” – **Male (IDM 007)**  “Then you will get other diseases like heart problems and kidney failure.” – **Male (IDM 013)** |
|  |  | Having T2D will reduce quality of life | “To me, if you want to have a good quality of life, try not to be diabetic lah.” – **Male (IDM 010)**  “Your brain won’t work properly, and you won’t do things actively (when you have diabetes).” – **Male (IDM 013)**  “Life becomes less enjoyable with diabetes.” – **Female (IDM 024)** |
|  |  | Injecting insulin means degree of T2D is serious | “Insulin is for those people whose disease is at a severe state.” – **Male (IDM 023)**  “When you take insulin, that means your sugar level is already very high…” – **Female (IDM 011)**  “I don’t like insulin. I feel that it’s something drastic.” – **Female (IDM 003)** |
|  |  | Explaining taking too much medication leads to ill health | “Taking too much tablets has a negative impact on a person’s body.” – **Male (IDM 012)**  “People say taking too much medicine is not good for you.” – **Female (IDM 021)**  “I’ve heard many people say that taking too many medicines can kill you at the end of the day.” – **Female (IDM 005)** |
|  |  | Ruminating over the effects medication on kidney function | “I fear that when I take too much medicines, I will get kidney problems…” – **Male (IDM 010)**  “I learnt that some medications affect your kidneys and whatnot.” – **Male (IDM 008)**  “…it’s the medication that actually destroys your kidneys and all.” – **Male (IDM 015)** |
|  |  | Taking rice influences T2D control | “When we eat rice, our sugar level goes up.” – **Male (IDM 012)**  “White rice is not good for you.” – **Female (IDM 020)**  “During fasting months, we take a lot of rice when we break fast. Then I notice that my blood sugar goes up.” – **Male (IDM 004)** |
|  |  | Thinking T2D can be controlled with lifestyle modifications alone | “I used to think that I can cure myself without medicine. That I could do things to reverse the disease…” – **Female (IDM 021)**  “I believe that exercise and diet can control the disease better than medication.” – **Female (IDM 011)**  “I think I can control my diabetes by consuming herbs and doing some hard work and sweating. I don’t think its necessary to take medicine.” – **Male (IDM 013)** |
|  |  | Believing daily activities are equal to exercising | “I feel that by my day to day activity, carrying things and going from shop to shop, that alone is better than jogging.” – **Male (IDM 018)**  “You know, when you sweep, cook, clean, wash, up stairs, down stairs, I feel like it’s exercise.” – **Female (IDM 005)**  “So the only exercise I do is housework.” – **Female (IDM 022)** |
|  | Knowledge | Describing mechanism for T2D (genetic, pancreas, insulin) | “I know that this disease is hereditary.” – **Female (IDM 020)**  “Diabetes is a disease that happens when your body doesn’t produce enough insulin lah.” – **Male (IDM 010)**  “From what I understand diabetes is this disease where our pancreas cannot produce insulin.” – **Male (IDM 023)** |
|  |  | Recognizing types of complications | “A few things I know, such as the leg can be cut off…” – **Male (IDM 009)**  “One of the complications is that your eyesight will get worse.” – **Male (IDM 016)**  “…and all our nerves will have issues…” – **Male (IDM 015)** |

**Definition:**

External environment – All outside factors or influences that impact self-management in of T2D patients

| Positive Disposition  *Positive inherent qualities of mind and character* | Positivity and Optimism | Having positive attitude towards insulin | “I don’t really miss my insulin, because I find insulin easy.” – **Female (IDM 006)**  “I take insulin, and I’m not embarrassed about it. Insulin is good for me.” – **Male (IDM 007)**  “I’m happy taking insulin. I even promote it to my friends.” – **Male (IDM 017)** |
| --- | --- | --- | --- |
|  |  | Claiming it takes little effort to comply to medication | “Actually it isn’t difficult to take the medication…” – **Female (IDM 024)**  “No, I face no problems in taking my medication.” - **Male (IDM 007)**  “To comply… it’s not that difficult lah, actually…” – **Male (IDM 004)** |
|  |  | Having no reservations or concerns about taking medication | “I just take it in my stride. So I now have no reservations about taking medication lifelong.” – **Male (IDM 008)**  “The medication doesn’t inhibit me in any way, and I have no issues taking it, because this is diabetes and this is how it is.” – **Male (IDM 010)**  “No, I have no negative thoughts about the medication.” – **Male (IDM 002)** |
|  |  | Having positive attitude towards modern medicine | “I feel that, these days medical science is so far advanced that we can do anything.” – **Male (IDM 012)**  “I have full confidence in the medication I take.” – **Male (IDM 017)**  “The people have invested millions into discovering these medications, its definitely good.” – **Male (IDM 008)** |
|  |  | Believing complications won’t happen if we take medication | “Yes, can. Most certainly can avoid complications if we take the medicine and follow the doctor’s instruction.” – **Male (IDM 002)**  “The chances of us getting complications depend on how well we take our medication. If I keep my sugar levels low, I shouldn’t get all these complications.” – **Male (IDM 010)**  “You can escape the complications if you control your blood sugar well.” – **Male (IDM 008)** |
|  |  | Expressing positive attitude towards medication compliance | “Ah, medication is routine, we have to take it.” – **Male (IDM 018)**  “I gotta take care of my health, so I take my medication.” – **Male (IDM 002)**  “Medication, after a while you get used to it.” – **Female (IDM 003)** |
|  |  | Dismissing that insulin injections are painful | “Insulin reacts faster, and it isn’t that painful.” – **Male (IDM 012)**  “I don’t think the pain is a problem. It’s just a needle, like when you are giving blood.” – **Male (IDM 010)**  “I was initially scared of injecting myself. After a week or two I got used to it, and it didn’t hurt.” – **Female (IDM 006)** |
|  |  | Feeling healthy despite having T2D | “As I’ve told you doctor, right now, I’m healthy. I’m a fighter.” – **Male (IDM 013)**  “I’ve been taking medication for so many years… I feel healthy. Yes, I feel healthy.” – **Male (IDM 017)**  “I don’t feel sickly at all. I feel like a normal person.” – **Female (IDM 022)** |
|  |  | Being confident can control disease | “It’s just diabetes. I can manage it if I want to.” – **Female (IDM 024)**  “I have full confidence that I can control my disease.” – **Male (IDM 017)**  “I know that I can control my diabetes.” – **Male (IDM 001)** |
|  |  | Adopting positive attitude towards disease | “I take my disease very positively. The main thing is, to take my medicine and control my blood sugar.” – **Male (IDM 008)**  “So even though I’m a diabetic patient, I’m full of life…” – **Male 9IDM 017)**  “Doesn’t matter who you are. All that’s left to do is accept it and move on.” – **Male (IDM 023)** |
|  |  | Feeling indifferent despite having T2D | “I don’t feel I’m sick or anything. For me life is just normal. Only that I have to take medicine.” – **Male (IDM 010)**  “Even though I am a diabetic patient, my way of life is still the same.” – **Male (IDM 017)**  “We don’t think about all the restrictions. We just carry on with our lives.” – **Male (IDM 013)** |
|  |  | Being confident have enough information about T2D | “Yes, I have all the information that I need to control my disease.” – **Male (IDM 012)**  “I understand what I need to do. The doctors don’t really have to tell me all again.” – **Male (010)**  “Yeah I feel I know enough.” – **Male (IDM 015)** |
|  |  | Right mindset | “I want to do better.” – **Female (IDM 005)**  “The good thing about me is, I’m a positive person.” – **Male (IDM 017)**  “If there is a problem with your attitude, then everything (patient’s control) will go wrong.” – **Female (IDM 011)** |
|  |  | Appreciating the value of life | “…don’t risk your life.” – **Female (IDM 005)**  “Love yourself.” – **Male (IDM 023)**  “…don’t appreciate the value of their life.” – **Female (IDM 011)** |
|  |  | Being true to self | “I want to be honest with myself, for my own good.” – **Male (IDM 015)**  “The fault of this (poor control) isn’t with the hospital. It’s with me.” – **Male (IDM 001)**  “People suddenly start controlling well just before check up. So the blood sugar will show a good result. But it won’t be the true result.” – **Male (IDM 010)** |
|  |  | Wishing to live longer | “I always tell my family I want to live a little longer.” – **Male (IDM 010)**  “I’ve changed and started controlling better because I want to live longer a bit.” – **Female (IDM 006)**  “I want to live longer, because I’m the breadwinner of the family.” – **Male (IDM 014)** |
|  |  | Being thankful because so far no complications | “So far, thankfully, no problems.” – **Male (IDM 002)**  “So for now, thankfully I have no complications. I can drink a little, smoke, run etc.” – **Male (IDM 010)**  “I feel that I’m lucky lah. I haven’t gotten all that (complication).” – **Male (IDM 008)** |
|  | Acceptance | Accepting the disease has occurred in us | I know I have diabetes, so I accept it.” – **Male (IDM 002)**  “I myself have accepted that I am a diabetic patient.” – **Male (IDM 012)**  “Then I told myself that this isn’t going to go away, so I might as well do what I have been doing and be happy.” – **Male (IDM 010)** |
|  |  | Accepting disease takes time | “It took me about 3 years to accept the fact that I was a diabetic patient.” – **Male (IDM 012)**  “At first I was really worried. Then after a few weeks or a month, I started to look at it practically.” – **Female (IDM 011)**  “So, after a year or so, I slowly accepted it.” – **Male (IDM 023)** |
|  |  | Accepting that fluctuations while on treatment is normal | “When you’re undergoing treatment, it’s like that. The readings won’t be stable.” – **Male (IDM 018)**  “Sometimes high, sometimes lower…like that.” – **Female (IDM 003)**  “Sometimes got high, sometimes got low.” – **Male (IDM 009)** |
|  |  | Having no problems taking insulin after initial fear | “After about a week of using it, I wasn’t afraid of insulin anymore.” – **Female (IDM 006)**  “I have no problems taking insulin.” – **Male (IDM 023)**  “Once we are used to it, we don’t really feel the phobia of taking insulin.” – **Male (IDM 015)** |
|  |  | Having no problems injecting in front of others | “No I don’t feel uncomfortable injecting myself in front of other people.” – **Female (IDM 021)**  “So far there is no problem injecting myself in front of others.” – **Male (IDM 023)**  “I’m not embarrassed to do so because I want to feel healthy.” – **Male (IDM 007)** |
|  |  | Resolving mental conflict whether to take medication or not | “Now it’s easier for me to take it, because I feel better after I take it.” – **Female (IDM 021)**  “Initially I didn’t want to take my medication, but now, I don’t have any conflicting thoughts anymore.” – **Female (IDM 005)**  “Initially I was really hesitant, but now I’m okay with my medication.” – **Female (IDM 024)** |
|  | Insight | Believing insulin is a potent alternative to oral medication | “Insulin actually reacts faster than oral medicine.” – **Male (IDM 012)**  “Compared to oral medicine, insulin is better.” – **Male (IDM 007)**  “I feel oral medications don’t do a lot for me. Insulin is the one that has the best effect.” – **Female (IDM 019)** |
|  |  | Changing perception about insulin after counseled by doctor | “Initially I was really worried about insulin. However, after the doctor advised me that insulin is better suited for someone like me, at this age and all, I felt much better.” – **Female (IDM 019)**  “…then the doctor advised me that it’s good for me, good for my pancreas and all that…” – **Male (IDM 016)**  “Once the doctor taught me about insulin, I felt okay about it.” – **Male (IDM 007)** |
|  |  | Knowing complications can occur if don't control T2D | “If I don’t take care of myself, this is what is going to happen (complications)” – **Female (IDM 021)**  “If I don’t change my poor control habits, I’ll end up like my family members (with complications).” – **Female (IDM 011)**  “Anybody can get complications if they don’t control.” – **Male (IDM 009)** |
|  |  | Wanting to control the disease | “Nowadays I feel like I really need to control lah.” – **Female (IDM 024)**  “I want to control, and prevent (complications)” – **Male (IDM 018)**  “I want the disease to be under control.” – **Male (IDM 004)** |
|  |  | Knowing medications help control the disease | “Taking medication is very important. Otherwise your blood sugar will keep increasing and your body will go haywire.” – **Male (IDM 008)**  “We already have this blood sugar. So medication helps keep the levels low.” – **Male (IDM 001)**  “Medications help. It helps control our disease.” – **Female (IDM 020)** |
|  |  | Knowing that timing is important in taking medications | “Of course. Timing is very important…” – **Male (IDM 004)**  “The medication will only work well if we take it correctly, at the right time.” – **Female (IDM 024)**  “…so yes, the timing is very important.” – **Male (IDM 023)** |
|  |  | Understanding necessity to be compliant to medication | “Taking medication is really important.” – **Female (IDM 006)**  “Yes I feel it’s important to take medication to control my diabetes” – **Male (IDM 009)**  “If you don’t take your medication you cannot be cured.” – **Male (IDM 013)** |
|  |  | Realizing the dangers of not taking medications | “If you don’t control, you are heading for disaster lah.” – **Male (IDM 014)**  “If you don’t take the medicine it will be worse.” – **Female (IDM 003)**  “If we don’t take medicine, we’ll just keep getting worse till we end up dead.” – **Male (IDM 015)** |
|  |  | Knowing T2D can only be controlled not cured | “It’s like I have been told. Diabetes cannot be cured.” – **Male (IDM 018)**  “If you want to completely cure diabetes, cannot lah. But we can get the sugar levels down.” – **Male (IDM 004)**  “This diabetes sickness definitely cannot be cured. I can only control it.” – **Male (IDM 002)** |
|  |  | Realizing that physical symptoms don't correlate with blood glucose levels | “When my sugar levels are high, I don’t feel sickly, you know.” – **Female (IDM 003)**  “Sometimes it shows, sometimes it doesn’t… it’s not the symptoms that show the blood sugar is high or not.” – **Female (IDM 011)**  “Even though we feel better, we cannot say that we are better, because we don’t know what is going on inside.” – **Male (IDM 016)** |
|  | Awareness | Knowing medications can cause side effects | “I know what medications can do. I know their side effects.” – **Female (IDM 011)**  “I know that there are side effects to this kind of medication.” – **Female (IDM 005)**  “Because we read and we know the side effects.” – **Female (IDM 020)** |
|  |  | Attributing cause of T2D to drinking sugary drinks | “I used to drink a lot of canned drinks, and that is one of the reasons I got this diabetes.” – **Male (IDM 004)**  “I wondered how I could get diabetes, because I worked physically. It’s perhaps the canned drinks I used to drink.” – **Male (IDM 013)**  “Then I used to have dinner with sugary drinks…” – **Female (IDM 019)** |
|  |  | Knowing types of food to eat in T2D | “You can’t take too much rice, you need to eat a lot of vegetables…” – **Male (IDM 002)**  “I had to change my entire food regime. I can’t take certain things, and other things I take in reduced amount.” – **Female (IDM 011)**  “You have to learn how to hate food. Food equals sugar.” – **Male (IDM 008)** |
|  |  | Attributing cause of T2D to high sugar consumption | “For those who are diabetic, our problem is sugar...” – **Male (IDM 017)**  “When I was in India, I used to eat sweets every day. You know Indians are famous for their sweets. That caused me to get diabetes.” – **Male (IDM 001)**  “If you are a sugar lover, then that will be what leads you to diabetes – **Male (IDM 010)** |
|  |  | Blaming lifestyle giving rise to T2D | “What causes diabetes? Diet, and lack of exercise.” – **Male (IDM 015)**  “In my opinion, diabetes is a disease that we bring on ourselves, due to what we put into ourselves.” – **Male (IDM 023)**  “I got diabetes because I wasn’t controlling my diet and wasn’t exercising.” – **Female (IDM 020)** |
|  |  | Knowing exercise and diet cannot replace taking medication | “You need to have all three things, exercise, diet and medicine. For diabetic people, all three must happen.” – **Female (IDM 020)**  “Diet and exercise can really help you control your blood sugar. But it cannot fully replace medication.” – **Male (IDM 016)**  “Your control depends on how well you control your food and do your exercise. But you still need the drugs lah. Still need the drugs.” – **Male (IDM 014)** |
|  | Vigilance | Being cautious due to fear of complications | “I’ve already gotten a carbuncle, so I’m kind of worried about the complications. So that makes me a little more cautious.” – **Female (IDM 024)**  “I’ve seen the complications of diabetes that has happened to other members of my family. So if I don’t change my habits now, I’ll end up like the other members of my family. I’ll end up with all the complications.” – **Female (IDM 011)**  “So now I am very cautious. I take supplements to help against the nerve cells dying. I am also very careful about small wounds on my body.” – **Male (IDM 012)** |
|  |  | Having T2D is not something to be taken lightly | “Over the years, diabetes can be quite fatal.” – **Female (IDM 011)**  “Diabetes cannot be cured. That’s why it’s dangerous.” – **Male (IDM 013)**  “Diabetes is a disease that is quite severe, for me.” – **Male (IDM 017)** |
|  |  | Needing to be more careful now have T2D | “I have to take better care of myself. I can’t just let my self bleed.” – **Female (IDM 021)**  “I have to take a lot of precautions in regards to my food intake.” – **Male (IDM 016)**  “You have to be careful once you get the disease. You need to control at all times.” – **Female (IDM 020)** |
|  | Coping Mechanism | Avoiding negative thoughts about prospect of disease | “I try not to think too much about the future. I don’t know how long I’m going to live, and I can’t control the future, so why must I be unhappy?” – **Female (IDM 021)**  “I tell myself that all these issues will not happen now. It’s still a long way off.” – **Female (IDM 006)**  “I try not to think about the negative things, and focus on the positive.”- **Male (IDM 013)** |
|  |  | Leaving it up to fate | “You can say you want to control all of this, but ether way you are going to die.” – **Male (IDM 012)**  “If I am to get the complications, then that’s just my fate…” – **Male (IDM 017)**  “I am old already. If I have to go, I’ll go.” – **Female (IDM 003)** |
|  |  | Believing that God determines fate | “I was a little upset that I got diabetes, but I believe that is my fate. It depends on God, doesn’t it?” – **Female (IDM 022)**  “As for complications, I leave that to God.” – **Male (IDM 016)**  “I don’t know, but for me, it all depends on God.” – **Male (IDM 009)** |
| Negative Disposition  *Negative inherent qualities of mind and character* | Denial | Finding difficulty to accept reality | “I rejected the doctor’s opinion that I have diabetes.” – **Male (IDM 012)**  “At that time, I couldn’t accept the fact that I was a diabetic.” – **Male (IDM 017)**  “I didn’t want to attend all these diabetic talks. I felt upset. I could not accept it.” – **Male (IDM 023)** |
|  |  | Finding it difficult to accept disease because had healthy lifestyle | “I am a sports man. I should not have gotten diabetes.” – **Male (IDM 002)**  “I used to be a policeman. My work used to be very physical. So I was in denial when they told me I had diabetes.” – **Male (IDM 023)**  “I work very hard. How did I come to develop diabetes?” – **Male (IDM 013)** |
|  | Pessimism | Frustrating have to take medication lifelong | “Compared to normal people, I have to take this medicine lifelong in order to survive.” – **Male (IDM 012)**  “That’s what affects me. The thought that I have to take it till I die.” – **Male (IDM 010)**  “I felt very disappointed when I found out I had to take medication lifelong.” – **Female (IDM 003)** |
|  |  | Getting fed up of taking medication | “Sometimes, after you’ve been taking medication for a long time, you get sick of it, you feel like you don’t want to take medicine. Let your body rest, you know, that feeling.” – **Male (IDM 012)**  “I take this medication daily, but sometimes I feel like I want to vomit taking all these pills. It’s like I can’t handle it anymore. Like I’m sick of it.” – **Female (IDM 021)**  “Sometimes I feel like I just don’t want to take the medication.” – **Female (IDM 006)** |
|  |  | Perceiving self to be sickly if take more medications | “Because I was told to take more medicine, I feel that I’m even more sick. More sick that’s why I have to take more medicine.” – **Female (IDM 003)**  “Taking medicine makes me feel less complete, compared to other people.” – **Female (IDM 021)**  “Strong people don’t need to take medicine. Normal people. Only sick people need to take medicine.” – **Male (IDM 007)** |
|  |  | Depressing T2D has occurred in self | “Initially I was really frustrated. I’ve worked at a ward before, I know all about diabetes I was a little upset.” – **Male (IDM 004)**  “I was really sad when I got diabetes, initially. I was a sportsman, I shouldn’t have gotten this.” – **Male (IDM 002)**  “To be honest, initially I was really depressed.” – **Female (IDM 021)** |
|  |  | Feeling restricted now that have T2D | “I feel really sad, because I cannot eat all the things that I want to eat.” – **Male (IDM 002)**  “There are a lot of things that you are restricted from doing when you are a diabetic patient.” – **Male (IDM 010)**  “Previously, before I was diabetic, I felt really active. Now that I have this disease, I feel very lethargic.” – **Male (IDM 009)** |
|  |  | Giving up on the disease | “Eventually you’ll think that you only have another 5 to 10 years, before you’re going to die, so what’s the point you struggle so hard.” – **Male (IDM 012)**  “We know there is no cure. So what’s the point of taking medication” – **Female (IDM 003)**  “Sometimes I give up hope for controlling the disease.” – **Female (IDM 021)** |
|  |  | Unwilling to live with complications | “I don’t want to live that kind of life (with complications)” – **Male (IDM 012)**  “I don’t want all these things (complications) to happen.” – **Female (IDM 011)**  “… I don’t want to die that kind of way (with a lot of complications), I want to die the normal way.” – **Female (IDM 005)** |
|  | Reservation | Having no choice but to take medications | “Whether or not I want to, I still have to take the medicine. Now that I have the disease, I have no choice but to take the medication.” – **Male (IDM 004)**  “I am a diabetic now, so I have no choice but to take medication.” – **Male (IDM 002)**  “I accept this disease. I got no choice. That means all my life I’ll have to take the drugs.” – **Male (IDM 014)**  “We are already diabetic. So we have to take the medicine no matter what.” – **Male (IDM 012)** |
|  |  | Having reservations about using insulin | “I don’t want to take insulin. I believe that there are a lot of side effects.” – **Male (IDM 004)**  “My wife is on insulin and I don’t want to be like her. She has to jab herself daily.” – **Male (IDM 008)**  “I don’t want to take insulin. That’s why I try very hard to control with my oral medicine. From what I see it looks quite painful.” – **Female (IDM 011)** |
|  |  | Having initial fear of using insulin | “Initially I was really scared of insulin. Scared of the pain.” – **Male (IDM 018)**  “To be honest doctor, initially, I really struggled with taking insulin.” – **Female (IDM 021)**  “It was only initially that I had the fear, the phobia of injecting myself…” – **Male (IDM 015)** |
|  |  | Feeling initial reluctance to commence on medications | “Yes, in the beginning I was a bit reluctant to take my medication.”- **Male (IDM 002)**  “Not anymore, doctor, but initially, around 20 years ago, I was really reluctant to take medicine.” – **Male (IDM 017)**  “At first I was mostly skeptical about the medication...” – **Male (IDM 014)** |
|  |  | Requesting to delay commencement of insulin | “Initially, when the doctor told me to start taking insulin, I told him to wait, and to let me try to control with oral medications alone, first.” – **Male (IDM 018)**  “When the doctor asked me to take insulin, I said ‘No, I don’t have any knowledge about this yet.’ So he gave me another appointment in 5 months.” – **Male (IDM 023)**  “I told my doctor I wasn’t interested in taking insulin. Tablets were okay, but I didn’t want insulin.” – **Female (IDM 011)** |
|  |  | Doubting the efficacy of medications | “Sometimes I question, whether or not the medicine really works…” – **Male (IDM 004)**  “I come in and for my check ups, after taking my medication. I can tell whether the medication is effective or not. And it seems to me that the medicine doesn’t really help, and that pharmaceutical companies are just selling it to make money.” – **Male (IDM 010)**  “Even if more medication is added, the readings are still high.” – **Female (IDM 003)** |
|  |  | Skipping medications due to side effects | “When I take my meds, one of the side effects are, I’ll get really drowsy. When that happens, I can’t go about my work. So I opt to not take meds.” – **Male (IDM 007)**  “… I feel really drowsy (when I take the medications), that’s why I don’t take them.” – **Male (IDM 013)**  “Yeah, the side effects make it really hard to take my medicine.” – **Male (IDM 008)** |
|  |  | Preferring oral medications over insulin | “I don’t like insulin. I’d much rather take tablets and try to bring down my sugar levels.” – **Male (IDM 010)**  “I think tablets are much better than insulin.” – **Female (IDM 003)**  “I’m aware of insulin. However I really don’t want to take it and that’s why I try to keep my sugar under control.” – **Female (IDM 011)** |
|  |  | Experiencing conflicting thoughts whether to take medications or not | “That’s the thing, doctor. I don’t know if I want to take the meds or not.” – **Male (IDM 015)**  “I feel like I really don’t want to take the medication. And then when the symptoms happen, I feel the need to take it.” – **Male (IDM 023)**  “I am supposed to take my meds at 12, but it’s already 1. Should I take my meds? Then I just decide not to.” – **Male (IDM 007)** |
|  |  | Hesitating to ask doctor out of fear of reprimand | “We don’t ask because we’re scared the doctor will scold us.” – **Female (IDM 003)**  “We are afraid that the doctor will think we’re challenging his knowledge.” – **Female (IDM 011)**  “We are afraid the doctor doesn’t have time for us and will get annoyed at our questions.” – **Male (IDM 015)** |
|  |  | Feeling unnecessary to increase strength of medication prematurely | “If there are no signs and symptoms, I feel there is no need to increase the dose of the medicine.” – **Male (IDM 004)**  “The medicine keeps increasing. That’s why I don’t like to take it.” – **Male (IDM 010)**  “The doctor, not only does he keep increasing my medicine, he gives me insulin on top of that. I feel it’s unnecessary” – **Male (IDM 007)** |
|  | Worries | Fearing complications might happen if don't take medication | “If I don’t take my medication, I’ll get a stroke!” – **Male (IDM 018)**  “I feel that if I don’t control well, there’s a possibility I’ll really get it (complications).” – **Male (IDM 004)**  “I understand what will happen if I don’t take my medicine.” – **Male (IDM 010)** |
|  |  | Wishing not to get any complications | “I’ve seen my brother get his fingers chopped off. I pray to god I don’t have to experience that.” – **Female (IDM 024)**  “Hopefully I don’t get any of those complications. If I can I try not to get it.” – **Female (IDM 022)**  “…your kidney fails, and you have to go for dialysis… I don’t want to go through all this, doctor.” – **Female (IDM 005)** |
|  |  | Fearing complications might happen in the future | “I’m worried about what will happen to me in future, with my sugar levels this high.” – **Male (IDM 018)**  “I worry about this disease affecting my kidneys, heart and all that.” – **Male (IDM 002)**  “These complications, I try not to get it. Because I’m scared, doctor, I’m really scared this may happen to me.” – **Female (IDM 022)** |
|  |  | Fearing complications will reduce quality of life | “We can even lose parts of our body, and that gives us an uncomfortable life.” – **Male (IDM 013)**  “I’ve seen how diabetes affects our life. My father went through it. I don’t want to go through that nightmare.” – **Female (IDM 005)**  “If we don’t control the sugar, it disease and complications takes control of us.” – **Female (IDM 011)** |
|  |  | Fearing complications | “My worries are my kidneys. I don’t want to have to go to the dialysis center every few days.” – **Male (IDM 002)**  “I have seen so many diabetic patients losing their limbs. That is my fear.” – **Male (IDM 012)**  “The main thing is heart problems. That’s why I do my best to keep my heart functioning well.” – **Male (IDM 001)** |
|  |  | Taking too much medication | “I’m worried that I’m taking too much medicine.” – **Male (IDM 010)**  “I’m worried because I’m eating a lot of medicine. People say taking too much medicine isn’t good for the body.” – **Female (IDM 021)**  “Sometimes I feel like I’m taking too much medicine.” – **Male (IDM 015)** |
|  |  | Fearing what might happen if don't take medication | “What will happen if I don’t take my medication? It’s a question I often ask myself.” – **Male (IDM 011)**  “If I don’t take my medication, I feel insecure, because I am sick and I don’t know what might happen.” – **Female (IDM 003)**  “I think about it. If I don’t eat my medicine, what will happen?” – **Male (IDM 004)** |
|  |  | Fearing pain from injecting insulin | “When it comes to insulin, I’m scared of the pain.” – **Female (IDM 019)**  “Most people don’t like insulin because of the pain of pricking.” – **Male (IDM 001)**  “Even when I see it, it looks quite painful.” – **Female (IDM 011)** |
|  |  | Fearing the use of insulin needles | “I don’t like needles. I don’t like to poke myself.” – **Female (IDM 003)**  “I’m afraid of the needles. I don’t like it.” – **Female (IDM 021)**  “I’m scared of the needle.” – **Female (IDM 024)** |
|  |  | Fearing increase in medication dose by doctor | “Every time I meet the doctor I’m worried he’ll increase the dose.” – **Male (IDM 004)**  “There’s no end to taking medicine. And every time the reading is high, the doctor adds more medicine.” – **Male (IDM 010)**  “And then the doctor said I need to increase the medicine. I don’t want to increase the medicine.” – **Female (IDM 003)** |
|  |  | Fearing reprimand | “I don’t dare tell the doctor a lot of these things, for fear of reprimand.” – **Female (IDM 019)**  “I don’t want to discuss these things with the doctor, because he’ll scold me for sure.” – **Female (IDM 024)**  “Some doctors are not patient friendly!” – **Female (IDM 020)** |
|  | Stigma | Classifying self as a sick person | “I feel like I’m not like other people. I don’t feel normal.” – **Female (IDM 019)**  “I am a sick person. Normal people don’t have to take medicine. Only sick people have to take medicine.” – **Male (IDM 009)**  “There is a weakness in your body. That’s why you have to support it with medicine.” – **Male (IDM 012)** |
|  |  | Being affected by negative perception of others about us | “So when people say things about us and our disease, we feel really down…” – **Male (IDM 004)**  “So when people say these discriminating statements to me, it affects my emotions, and makes me want to miss my medications.” – **Female (IDM 006)**  “All these words (negative statements about the disease) really freak you out…” – **Female (IDM 005)** |
|  |  | Feeling will be discriminated if know T2D present | “I’ve seen the reaction others have had when they find out I’m diabetic. It’s as if I’m going to die tomorrow.” – **Male (IDM 023)**  “When they find out that a person has diabetes, they will look down on them.” – **Female (IDM 011)**  “My job (tourist driver) depends on people being comfortable around me. If they find out I’m diabetic, they will think that their driver is sick.” – **Male (IDM 017)** |
|  |  | Keeping the diagnosis of T2D confidential | “I don’t tell people I’m diabetic.” – **Female (IDM 003)**  “I don’t tell people I have diabetes because I want to preserve my dignity.” – **Female (IDM 021)**  “I don’t want people to know I have this sickness.” – **Female (IDM 005)** |
|  |  | Feeling embarrassed having T2D | “First I felt imperfect. Then I felt embarrassed about myself…” – **Female (IDM 021)**  “I felt really embarrassed, that a young guy like myself could get diabetes.” – **Male (IDM 007)**  “Sometimes I feel ashamed as well…” – **Female (IDM 019)** |
|  |  | Excusing self in order to inject insulin | “No I don’t take my insulin in public. I prefer doing it in private.” – **Male (IDM 012)**  “At my work place I wait till I’m alone before I take my insulin.” – **Female (IDM 011)**  “I prefer to take insulin alone. Even if its just around my wife and children, I prefer to take it alone.” – **Male (IDM 023)** |
|  |  | Fearing what others might think when know injecting insulin | “When I take insulin in front of my relatives, there’ll definitely be those that make fun of me.” – **Female (IDM 006)**  “When I take insulin, those people may think I’m injecting drugs into me.” – **Male (IDM 009)**  “For those that know insulin, it’s okay. For those that don’t know, they’ll get worried and wonder what I’m injecting into my body.” – **Female (IDM 019)** |
|  |  | Feeling depressed and embarrassed have to use insulin | “I have to hide and take my medicine. Because I’ve seen the reaction people have when I eat pills in front of them…” – **Female (IDM 021)**  “I get uncomfortable when people see me taking medicine in public.” – **Male (IDM 009)**  “Sometime I just don’t want to take tablets in front of people. Then they will comment.” – **Female (IDM 005)** |
| Self Management Practices  **Helpful**  *Positive actions taken to manage symptoms, treatment and lifestyle changes*  **Unhelpful**  *Negative actions taken to manage symptoms, treatment and lifestyle* | Self Efficacy | Complying to prescribed medication | “My children always see me taking my medicine regularly. So they know I automatically will take.” – **Male (IDM 017)**  “I really follow my medication as prescribed.” – **Female (IDM 019)**  “What the doctor has prescribed, I just follow.” – **Male (IDM 002)** |
|  |  | Bringing medication along when out of home | “If I happen to go outstation, I bring my medicine along with me.” – **Female (IDM 019)**  “Nowadays, the medicine is always in my bag.” – **Female (IDM 022)**  “I keep my medicine in a box, and I keep it with me at all times.” – **Male (IDM 010)** |
|  |  | Setting reminder on phone to improve compliance | “…Otherwise, what I do is I set a notification on my phone.” – **Male (IDM 018)**  “I use my phone as a reminder for my medicine.” – **Male (IDM 014)**  “My phone is an indicator that it’s time to take meds.” – **Male (IDM 010)** |
|  |  | Preventing missed dose of oral medications by storing in containers | “I keep all my medicine in a little box.” – **Male (IDM 010)**  “For myself, I keep my medicine in a small box…” – **Female (IDM 003)**  “I organize my medicine into little boxes…” – **Female (IDM 021)** |
|  |  | Making medication taking a routine activity | “The medicine is part of my life, doctor.” – **Male (IDM 013)**  “Medicine is a daily routine. We cannot forget.” – **Male (IDM 016)**  “If you are a routine kind of guy, then you will realize that something is missing if you don’t take your medicine.” – **Male (IDM 012)** |
|  |  | Reminding self to take medication | “When I forget my medicine, I will remind myself to take it later.” – **Male (IDM 010)**  “I keep reminding myself and try not to forget to take the medicine.” – **Female (IDM 003)**  “I keep telling myself I need to take the medicine properly.” – **Female (IDM 005)** |
|  |  | Adhering to proper timing when taking medications | “I have a timetable of when I should take the medicine.” – **Male (IDm 018)**  “I know that after I take my tablet, half an hour later, I have to have my meal. So I do that.” – **Female (IDM 020)**  “Medication, I try to be on time.” – **Male (IDM 017)** |
|  |  | Self-monitoring blood glucose levels | “I usually like to do that. Check my blood sugar before and after taking medicine.” – **Male (IDM 004)**  “I check my sugar maybe once a week or once every 2 weeks.” – **Male (IDM 012)**  “I check it (blood glucose levels) everyday. Every day, first thing in the mornings.” – **Male (IDM 008)** |
|  |  | Recalling proper insulin technique and utilization | “You have to change the needle regularly…” – **Male (IDM 009)**  “It’s (insulin) more effective if I take it in the stomach.” – **Female (IDM 006)**  “Need to shake before pressing…” – **Male (IDM 018)** |
|  |  | Practicing foot care | “I have to make sure my foot is clean, and I ask the doctor to check and make sure…” – **Male (IDM 002)**  “When I walk and all, whenever I do anything I am very careful about my feet.” – **Female (IDM 005)**  “I wear slippers indoors, because I’m afraid of small rocks and stones.” – **Female (IDM 024)** |
|  |  | Improving self care by using feedback from glucometer | “I check my own blood sugar, and draw motivation from that.” – **Male (IDM 018)**  “When I check my sugar level, and it goes up to about 9 or 10, then I will do something to bring it down.” – **Male (IDM 010)**  “I will go and check, and when it is high, I will backtrack and question what I have eaten or done to cause it to be so high.” – **Female (IDM 011)** |
|  |  | Following up for T2D check ups | “And so, till this day I continue going for all my check ups.” – **Male (IDM 018)**  “I have checkups that I follow regularly.” – **Female (IDM 011)**  “I still follow my 4 months once control visits here (at the Klinik Kesihatan). – **Female (IDM 005)** |
|  |  | Needing to consult doctor if in doubt | “I’m afraid to change the dose on my own. So I’ll ask the doctor. I don’t have that knowledge. The doctor does.” – **Male (IDM 017)**  “I found out that all these other remedies are dangerous. In order to cure the disease, you need the doctor…” - **Male (IDM 013)**  “Whenever something small happens, I always say, go to the doctor and get a check up.” – **Male (IDM 001)** |
|  |  | Preventing complications by taking medications | “The two complications I’m most worried about is kidney failure and stroke. So by taking this medication, I hope that I can avoid these two.” – **Male (IDM 017)**  “As a diabetic patient we have to take our medicine. If we don’t do it now, later on we’ll face all sorts of problems.” – **Male (IDM 013)**  “I believe that medicine helps me control my sugar. If I control my sugar, everything will be alright. I won’t get any complications.” – **Male (IDM 002)** |
|  |  | Needing to control diet | “We must control our diet.” – **Male (IDM 001)**  “Food intake you must control.” – **Male (IDM 017)**  “Nowadays I really control my diet lah. I avoid all those things that I’m not supposed to eat.” – **Male (IDM 023)** |
|  |  | Following dietary advice | “I’m implementing changes in my diet.” – **Female (IDM 003)**  “I’ve drastically reduced the sugar I take in.” – **Female (IDM 006)**  “I know how to control my food and keep myself under control.” – **Female (IDM 005)** |
|  |  | Reducing the intake of rice | “When I eat out I ask them to put less rice for me.” – **Female (IDM 003)**  “…or I take a very small quantity of rice.” – **Female (IDM 005)**  “I reduce my rice intake. For example, if I take one meal of rice, my next meal I don’t take rice. I take something else.” – **Male (IDM 016)** |
|  |  | Choosing more vegetables | “Nowadays I have developed a preference towards raw vegetables, like salads and whatnot.” – **Female (IDM 019)**  “I control my food by taking less rice and more vegetables.” – **Female (IDM 020)**  “I eat vegetables, it is good for me.” – **Female (IDM 011)** |
|  |  | Using dietary measures to bring down elevated blood glucose | “I control my food to control my sugar level.” – **Male (IDM 012)**  “I control my food. I don’t simply eat anything. And when I check my blood, it’s 6, or 5.5.” – **Male (IDM 010)**  “I told myself to cut out all the sugary foods, so that I can bring my body back to health.” – **Male (IDM 013)** |
|  |  | Wishing to bring down blood glucose to optimal level | “I want to bring my blood sugar down. I want it to be 5 or 6 lah.” – **Male (IDM 014)**  “I feel that I can really control it, and I want to bring my sugar down from it’s current level.” – **Male (IDM 017)**  “My blood sugar is 7.7. I wish to reduce it further.” – **Male (IDM 008)** |
|  |  | Exercising to control blood glucose | “I try to reduce the sickness by doing exercise.” – **Female (IDM 003)**  “As of late I’ve started to do a little jogging, and it’s brought down my sugar to 7.7.” – **Male (IDM 008)**  “At home I exercise and feel quite good.” – **Male (IDM 018)** |
|  | Rational | Trusting information from internet | “In my experience, some of the things that influence my diabetes are articles that I read on the internet.” – **Male (IDM 015)**  “See that’s the thing, doctor. Because I don’t have the knowledge, I’ve got to explore. Read articles on the internet. And so I read these things on the internet, articles written by medical websites or other doctors.” – **Male (IDM 023)**  “So I just type the name of the medication in the internet and I can see everything about it.” – **Male (IDM 001)** |
|  |  | Choosing what information is useful to us | “When people tell me things, it’s not that I accept everything. I decide what to accept.” – **Female (IDM 011)**  “When they (people) tell you something, you need to think for yourself, whether it can be accepted or not.” – **Male (IDM 016)**  “I listen to the information, but I see first. I balance everything, and decide which I want to follow.” – **Female (IDM 020)** |
|  |  | Believing evidence based information | “Newspapers always come with the latest information, doctor. Information about researches done overseas and everything.” – **Male (IDM 002)**  “I educate myself and do my own research.” – **Male (IDM 014)** |
|  |  | Being skeptical about CAM | “If it isn’t western medicine, that means it (CAM) comes from leafs and herbs. And there’s no proof that it works.” – **Male (IDM 010)**  “Traditional medicine, as in the products sold out there? To me that’s rubbish lah.” – **Female (IDM 021)**  “Those village remedies, I strictly don’t take.” – **Female (IDM 011)** |
|  |  | Dismissing the need for CAM when conventional management effective | “When the medicine I’ve been given works, I don’t see why I need to go and take anything else.” – **Female (IDM 021)**  “When I think about it, it’s much better for me to follow the medication provided by the hospitals.” – **Male (IDM 015)**  “Traditional medicine is useless. You’re much better off with medicine from the hospital.” – **Male (IDM 007)** |
|  |  | Being suspicious about CAM leading to side effects | “I also don’t know whether taking all these (CAM) will lead to side effects or not.” – **Male (IDM 010)**  “Traditional medicine, we only have word of mouth to go by. But if anything happens, that’s it.” – **Female (IDM 006)**  “No, traditional medicine should not be consumed. You never know, it could cause you heart problems and you’ll suffer.” – **Male (IDM 007)** |
|  |  | Being skeptical about efficacy of CAM in self | “I don’t know whether the traditional medicine I’m taking works on me or not.” – **Male (IDM 004)**  “I used to take for a few months, but I didn’t feel any difference at all.” – **Male (IDM 023)**  “I tried it and I felt my body didn’t respond well to me. So I stopped it.” – **Female (IDM 024)** |
|  |  | Trusting doctor's advice or instruction | “The guy who decides should be the doctor. He’s the person that knows best.” – **Male (IDM 008)**  “What the doctor says, I’ll do. I will follow the doctors procedure.” – **Female (IDM 019)**  “I will take my medication according to the advice of the doctor.” – **Male (IDM 017)** |
|  |  | Knowing it is up to us whether to accept comments of others | “People tend to say a lot of different things, but it’s up to us whether we listen to it or not.” – **Male (IDM 004)**  “People say all sorts of things; try to get me to do all sorts of things, none of that influences me.” – **Female (IDM 005)**  “My friends say a lot of things, telling me I’m weak or I have a weakness. None of that influences me.” – **Male (IDM 016)** |
|  |  | Trusting doctor's advice is completely up to patient | “If the patient wants to take the medicine, he’ll take it. If he doesn’t want to take it, he won’t. The patient will choose.” – **Female (IDM 020)**  “For me it’s not an issue. The advice, if I can follow, I follow. If I can’t follow, I don’t follow lah.” – **Male (IDM 009)**  “Whether or not the patient is willing to accept the advice is up to them.” – **Male (IDM 018)** |
|  | Dietary restraint | Restricting sugar intake | “I’ll tell them to put less sugar in my tea.” – **Male (IDM 002)**  “Nowadays when I order my tea I immediately ask for less sugar, or no sugar.” – **Male (IDM 012)**  “I am not taking sugar anymore. For my drinks I’m using sweeteners.” – **Female (IDM 003)** |
|  |  | Being able to still enjoy food by reducing amount previously consumed | “So these days I take my meds, and I eat what I want. But not a lot. In small amounts, just to taste.” – **Female (IDM 024)**  “Everyone asks whether I can take some sweet things and all. I can. For example, if I go to a birthday party, I’ll take the cake, but only a very small piece.” – **Female (IDM 020)**  “I had to change my entire diet. Certain things I can’t take, some I take in smaller amounts.” – **Female (IDM 011)** |
|  |  | Practicing restraint when consuming food | “You can choose what you want to eat. You don’t have to put all the junk into your mouth.” – **Female (IDM 003)**  “When there are gathering, I politely refuse, take my plain water and a small amount of food, and eat.” – **Male (IDM 023)**  “I really control my diet. Vegetables and fish. I controlled with that type of food.” – **Female (IDM 019)** |
|  |  | Avoiding sugary beverages | “I don’t have the urge to take sugary drinks anymore.” – **Male (IDM 012)**  “I tell the people who are unaware that I’m diabetic, not to give me sweet drinks.” – **Female (IDM 020)**  “I don’t drink carbonated drinks.” – **Male (IDM 017)** |
|  | Responsibility | Being proactive about disease | “I actively learn, maybe by asking a lot of questions.” – **Male (IDM 004)**  “I do research for myself about insulin and all that.” – **Male (IDM 014)**  “Each time the doctor changes or increases my medication; I’ll make it a point to ask him about the drug I’m taking.” – **Female (IDM 011)** |
|  |  | Knowing discipline is important in T2D management | “You have to be disciplined to take the medicine properly. Then you will see the results.” – **Male (IDM 012)**  “I am a person with certain principles. If this is something I have to do, I do it. No matter what.” – **Female (IDM 011)**  “We have to train ourselves, and really take a lot of effort to learn to comply to the medicine.” – **Male (IDM 013)** |
|  |  | Knowing its patient's responsibility to comply with medication | “It’s my duty to take the medicine.” – **Male (IDM 016)**  “If we are responsible to ourselves, we’ll take the medicine as per regulation.” – **Male (IDM 015)**  “We need to take that medicine, we need to be responsible…” – **Female (IDM 021)** |
|  |  | Taking responsibility to look after their health | “You have to be serious (about health) now that you have diabetes…” – **Male (IDM 008)**  “I have to take care of myself.” – **Male (IDM 002)**  “It is definitely the individual who’s responsible for their disease.” – **Male (IDM 012)** |
|  |  | Maintaining health by taking medication | “I take the disease very positively. The most important thing is to take the medicine to control my blood sugar.” – **Male (IDM 008)**  “If I want to survive, I have to continue taking medicine.” – **Male (IDM 002)**  “I take my medication to keep my sugar levels low.” – **Male (IDM 010)** |
|  |  | Maintaining health so that can live longer | “One of the reasons I do this (control) is because I think of the long run.” – **Male (IDM 018)**  “I follow the doctor’s advice, and if I’m healthy, then I can stay a little longer here (on earth).” – **Male (IDM 002)**  “If I die, I die. But I try to prolong my life. To me that’s good enough already.” – **Male (IDM 017)** |
|  |  | Staying healthy to improve quality of life | “If I want my legs, fingers and eyes to be fine, I have to take my medicine. That’s all.” – **Female (IDM 005)**  “Everyone wants a good, healthy life. And I have this diabetes. If I don’t control it, I could go blind…” – **Male (IDM 014)**  “We try to control because we want to be better. Otherwise we will be very sick.” – **Female (IDM 003)** |
|  |  | Being healthy so can support the family | “I need to work on my health because I need to take care of my family.” – **Male (IDM 002)**  “I don’t want my child to be motherless.” – **Female (IDM 006)**  “My generation tends to think this way. We think that we have to be strong for our family. So if we have any sickness, we would take the medicine, because we have a responsibility (to family).” – **Female (IDM 021)** |
|  |  | Managing disease independently | “I manage my own disease (no involvement of family members).” – **Male (IDM 012)**  “No my family doesn’t need to prompt me to take medication.” – **Male (IDM 010)**  “My family, my children don’t advise me because they don’t know enough about diabetes.” – **Male (IDM 023)** |
|  |  | Maintaining health to avoid being a burden to others | “I must take care of my disease so that I won’t be a burden to my family and my people.” – **Male (IDM 002)**  “I don’t want to trouble my children to have to send me to and fro from the hospital.” – **Male (IDM 015)**  “If I don’t take the medicine, I’m troubling my family members.” – **Male (IDM 013)** |
|  |  | Persevering with medication despite side effects | “Even today the side effects are there. But I’m not bothered.” – **Female (IDM 011)**  “Even though the metformin gives me trouble, I still continue to take it, because the doctor asked me to.” – **Female (IDM 006)**  “I feel all these (side effects), but I still take my medicine. I never stop. I don’t want to stop.” – **Female (IDM 005)** |
|  |  | Complying to medication despite no physical symptoms | “Yes, I believe we should take our medicine and come for check ups, even if we feel physically well.” – **Male (IDM 002)**  “Even though I feel really healthy, I’ll still take my insulin.” – **Male (IDM 006)**  “Even if I feel healthy, I’ll continue with the medication.” – **Male (IDM 017)** |
|  |  | Missing medications infrequently | “…but about 90% of the time, I don’t miss my medicine.” – **Female (IDM 011)**  “I have forgotten before, but it’s a very rare occurrence.” – **Female (IDM 006)**  “Once in a while I’ll forget.” – **Male (IDM 009)** |
|  | Neglect | Missing medication because of procrastination | “Why I miss my medicine? I’m not sure if this is the right word for it, procrastination? Yeah.” – **Male (IDM 010)**  “I put off taking medicine when I should, then later on, when I should take, I’ll be on an empty stomach. Then I don’t know whether to take or not.” – **Female (IDM 011)**  “Maybe sometimes people feel that they don’t need to take the medicine today, I’ll take it tomorrow…” – **Female (IDM 020)** |
|  |  | Confessing having skipped medications | “There have been times where I don’t take my medicine.” – **Male (IDM 004)**  “I’m also not that good at complying with my medicine.” – **Male (IDM 015)**  “Previously I never used to take my medicine.” – **Male (IDM 007)** |
|  |  | Attributing forgetfulness to take medications to old age | “I forget sometimes. You know, especially us seniors, we’re prone to forget.” – **Male (IDM 018)**  “Forgetfulness is an issue (in taking medication). Especially amongst people who are older.” – **Female (IDM 011)**  “People who get older are bound to forget their medications.” – **Male (IDM 016)** |
|  |  | Reminding self to take medication as appointment approaches | “The closer you come to the appointment date, you will start to worry and start taking your medicine.” – **Male (IDM 012)**  “When it’s nearer the appointment date, only then will I start taking my medicine properly, because I don’t want to get scolded by the doctor.” – **Male (IDM 015)**  “If I’m being honest, there was a time where I would only control when it was close to the appointment date.” – **Female (IDM 019)** |
|  |  | Having no awareness about the dangers of high sugar diet | Those days, I used to eat a lot of sweets in India. For 14 years. There was no awareness at all about diabetes back then.” – **Male (IDM 001)**  “It was a habit formed in my younger days. Staying overseas, I drank Coca-Cola 100% of the time.” – **Male (IDM 023)**  “Coca-Cola used to be a daily affair for me. Back then I didn’t know about the effects of such drinks.” – **Male (IDM 018)** |
|  |  | Confessing not doing much exercise | “For me, I don’t exercise.” – **Male (IDM 018)**  “I don’t exercise very much.” – **Male (IDM 010)**  “The thing about me is that I hardly do exercise.” – **Female (IDM 024)** |
|  |  | Missing medication due to forgetfulness | “Sometimes I forget. I leave the pill box at home.” – **Male (IDM 008)**  “I rarely miss, unless, for example, certain days I forget.” – **Female (IDM 019)**  “I almost always take it. Unless I forget.” – **Female (IDM 022)** |
|  |  | Having lackadaisical attitude towards T2D management | “There are many reasons why diabetes is a norm. For example the craving for food and the ‘don’t care’ attitude.” – **Female (IDM 011)**  “I take the medicine from the doctor, but when I go home, I go back to my old lifestyle…” – **Male (IDM 015)**  “You can control your disease by taking medication, dieting and exercising regularly. However, I don’t do it.” – **Male (IDM 009)** |
|  |  | Missing or skipping medication (deliberate) | “I didn’t take that medicine at all, because I know my health was okay.” – **Male (IDM 013)**  “Some medicine I’ve been asked to take daily, I’ll take once every two days.” – **Male (IDM 010)**  “Just like that, one day I could not take it anymore. I didn’t want to take any of my medicine. I went like that for two days.” – **Female (IDM 011)** |
|  |  | Violating dietary restriction | “I don’t follow the doctor’s advice especially when it comes to food.” – **Male (IDM 002)**  “My diabetes isn’t very well controlled, mostly due to my diet.” – **Male (IDM 015)**  “In the middle of the night, when I’m driving my lorry, if I feel sleepy I’ll eat whatever’s convenient, mostly stall food.” – **Male (IDM 007)** |
|  |  | Being ignorant about the disease | “When I was diagnosed with diabetes, I knew I had a disease, but I knew nothing about the disease.” – **Female (IDM 024)**  “The doctors told me I had diabetes, but I didn’t understand. To me, I was healthy.” – **Male (IDM 013)**  “I didn’t know anything about this disease, and I didn’t understand anything. I didn’t bother going out there to find out ether.” – **Female (IDM 021)** |
|  |  | Failing to take medication according to proper timing | “I take my medication. It’s just that sometimes, due to my lifestyle, the timing tends to be erratic.” – **Male (IDM 004)**  “For example, I am supposed to take a tablet in the morning, but sometimes I don’t have my breakfast, and so I forget about the medication. Then later when I take it, it isn’t according to the proper time.” – **Female (IDM 021)**  “Sometimes I sleep through the time I’m supposed to take medicine. Now I’m not working, so my sleeping hours are irregular.” – **Male (IDM 013)** |
|  |  | Blaming self for lapses in T2D management | “Sometimes, it’s my mistake. I don’t follow the doctor’s advice. Especially in regards to food.” – **Male (IDM 002)**  “I feel that, if ever I were to get any complications, like my foot getting amputated, for example, it would be my fault. It would be because I didn’t control properly.” – **Male (IDM 023)**  “Nobody influences my medicine intake. If I fail to take my medicine, then that’s my own fault.” – **Female (IDM 005)** |
|  |  | Confessing having missed medication | “During my younger days, I never used to take my medicine. I always missed it.” – **Female (IDM 006)**  “To be frank, I miss my medicine as well sometimes. Especially at night, when I have to go somewhere.” – **Female (IDM 022)**  “Yeah as I’ve mentioned, I sometimes forget to take my medicine.” – **Female (IDM 019)** |
|  |  | Being unaware of having T2D | That time, if I had not gotten dengue and gotten my tests, I would not have known I had diabetes.” – **Male (IDM 002)**  “I only discovered I had diabetes when I went for my check up. I wanted to attend a course to learn sales, so the requirement was to get a medical checkup done.” – **Male (IDM 007)**  “Me discovering I had diabetes was coincidental. I came in for my back pain. Only then did I realize I had hypertension and diabetes.” – **Female (IDM 005)** |
|  |  | Lacking discipline to follow T2D management advice | “Believing (diabetes and methods to control) is one thing. Actually performing it is another thing altogether.” – **Male (IDM 010)**  “Sometimes because of greed, I tend to eat extra.” – **Female (IDM 011)**  “It’s just that, as a diabetic patient, I’m not very disciplined.” – **Female (IDM 024)** |
|  |  | Complying to T2D management once complications set in | “I only started taking my diabetes seriously after I got married. My eyesight had become very bad. I could not see.” – **Female (IDM 006)**  “My own wife only started taking her medicine after her foot got gangrene.” – **Male (IDM 023)**  “It was only that time when I was admitted for my carbuncle did I start controlling well.” – **Female (IDM 024)** |
|  |  | Thinking about not complying due to no physical symptoms | “Yes, during the initial stages of my disease, I too thought that I didn’t have to take medicine once I felt healthy.” – **Male (IDM 004)**  “Sometimes I try not to think about it. If I feel healthy, then I just enjoy myself, and I think that helps.” – **Male (IDM 007)**  “I tend to only control when I feel sick. When I feel healthy, I go back to my daily routine.” – **Female (IDM 024)** |
|  |  | Realizing T2D too late when extreme symptoms arise | “I didn’t know I had the disease until I started feeling really sick and weak.” – **Male (IDM 018)**  “That time, I felt really giddy and thirsty. Then my boss’s brother asked me to go check my sugar. And then I was diagnosed with diabetes.” – **Male (IDM 002)**  “The thing about diabetes is that you can’t really feel it until much later on when you start to feel really ill.” – **Female (IDM 022)** |
|  |  | Violating proper insulin technique | “If you don’t change the insulin needle regularly, when you poke, it feels very painful.” – **Male (IDM 009)**  “They advised me to regularly change my insulin needles, but, sorry to say doc, I don’t really do that.” – **Female (IDM 024)**  “Back then, I didn’t take my insulin properly. I never shake the bottle, and I never used to press first before injecting.” – **Male (IDM 018)** |
|  |  | Failing to disclose clinical information about self to doctor | “I know it’s my fault, but sometimes I don’t really tell the doctor things about my disease…” – **Male (IDM 004)**  “Sometimes we don’t tell the doctor everything because we are afraid the doctor may scold us.” – **Female (IDM 003)**  “I faced all these issues, but I’ve never really told the doctor. I know the doctor has his own job, and he will advise me to take the medicine. That’s fixed. So I’ll follow what I can.” – **Male (IDM 007)** |
|  |  | Detecting predominantly high blood glucose levels with glucometer | “Most of the time my glucose is really high when I test it.” – **Male (IDM 004)**  “Every time I check my (blood glucose) readings, the results are bad.” – **Female (IDM 005)**  “When I come and check it, the readings are almost always high.” – **Female (IDM 019)** |
|  |  | Attributing T2D to uncontrolled food consumption | “My diet isn’t very good, doctor. I tend to eat a lot of ice creams. And for dinner I usually drink sugary drinks.” – **Female (IDM 019)**  “Nowadays we have all the junk food and fast food that cause this disease.” – **Male (IDM 001)**  “As Malaysians, we are sugar lovers. We overindulge in sugar. So in the long run, we will reap the effects of our bad habits.” – **Male (IDM 012)** |
|  |  | Frustrating having to follow diet restrictions | “Food restrictions are one of the main reasons why diabetes control fails. When a normal person can eat that food, why can’t I? I didn’t ask for this disease.” – **Male (IDM 012)**  “If you are a person who likes to eat, then controlling this disease is really hard. When I go to a function, I feel so restricted. There is so much good food, but I can’t eat.” – **Male (IDM 010)**  “Of course the doctor is right in saying all those things that we are not supposed to eat. But it sometimes just stresses me out.” – **Male (IDM 023)** |
|  |  | Expressing difficulty in controlling diet | “I feel that controlling the diet is really difficult.” – **Male (IDM 002)**  “When you sit down with the dietician, all the advice the dietician gives you is good and for your own good. But sometimes it’s just not practical.” – **Male (IDM 012)**  “Controlling my diet is difficult, doctor. Especially since, since young, I’ve been raised to eat heavy food. Now you’re telling me to change and eat a few slices of bread and oats? I can’t even sleep because of that.” – **Male (IDM 015)** |
|  |  | Violating dietary restrictions due to social pressure | “I try to inform them (friends/guests) in advance about my diabetes. However if they’ve given me the food or sugary drink, I cannot refuse ether.” – **Female (IDM 020)**  “Then I’ll make a stop on the way to deliver goods, and have a drink with my friends, and drink and eat junk food.” – **Male (IDM 007)**  “The food is the difficult part. When 2 or 3 of us gather at the hawker stalls, it’s really difficult to maintain dietary control.” – **Male (IDM 015)** |
|  |  | Consuming forbidden or excess food despite knowing | “I’ve spoken to the dietician and I know about the portions I should be eating. But sometimes, I just feel hungry, doctor. And when that happens, I’ll eat till I’m full.” – **Female (IDM 019)**  “Sometimes, because of greed, we eat more than we should.” – **Female (IDM 011)**  “Sometimes, when we’re gathered with family, and all the food is laid out before us, we get tempted and we give in.” – **Male (IDM 023)** |
|  |  | Condoning violations in dietary restrictions (infrequent) | “I can control well for most things. I take my medicine and I cut down all sugary stuff. But the things I cannot give up are rice and coffee.” – **Female (IDM 006)**  “Once in a while, doctor, when we eat out, the temptation is there. So I indulge.” – **Male (IDM 017)**  “At times I binge eat. I cannot really control myself.” – **Male (IDM 008)** |
|  | Experimentation | Changing and continuing medication dose (not as prescribed) | “I’ve done some experiments with my medicine. And I’ve concluded that I’ll keep taking my medicine, but at a lesser dose.” – **Male (IDM 004)**  “To tell you the truth, this one medicine, doctor prescribed 2, I only take 1 and a half.” – **Male (IDM 010)**  “Before this, I have tried to lower my dose. I’ve never been brave enough to try increasing it.” – **Female (IDM 021)** |
|  |  | Seeing increase in blood glucose levels after altering/skipping dose | “I realized that I should not experiment with my medication, when I saw that my blood sugar was not improving.” – **Male (IDM 012)**  “So I play around with the dose and sometimes skip medicine for a day. Then when I check, I see an increase in my sugar levels.” – **Female (IDM 011)**  “When I take the full 500mg I feel dizzy. So I reduce the amount. Then when I meet with my doctor, my readings all go up.” – **Male (IDM 007)** |
|  |  | Taking CAM | “I take some traditional medicines, like herbs.” – **Male (IDM 004)**  “I used to go to India once a year. When I’m there I take the Indian traditional medicine.” – **Male (IDM 002)**  “What I do is I chew on this ‘neem’ leaf.” – **Male (IDM 012)** |
|  |  | Setting time interval for consuming CAM after medications | “There is no harm in taking traditional medicines, but you must leave a gap between the hospital medicine and the herbal medicine.” – **Male (IDM 001)**  “Yes if I take my herbal medicine, I’ll wait one hour before taking my normal medicine.” – **Male (IDM 016)**  “If you take traditional medicine you must leave a gap of at least half an hour before taking your hospital medicine.” – **Male (IDM 009)** |
|  |  | Adding CAM to current treatment (not replacing) | “So I take this traditional medicine, more to supplement the treatment.” – **Male (IDM 004)**  “When taking traditional medicine, you should not stop the hospital medicine. You must take both.” – **Male (IDM 002)**  “No, I’m not replacing my western medicine. I take cinnamon and I take my normal medicine.” – **Female (IDM 020)** |
|  |  | Replacing medication with CAM (entirely - skip) | “I once tried, for a period of time, to abandon my modern medicine and take herbal medicine.” – **Male (IDM 004)**  “I replaced metformin with herbal medicine, because metformin gave me some sort of giddiness.” – **Male (IDM 013)**  “Sometimes I’ll try the remedies my friends give me. I’ll take the traditional medicine exclusively for a few days.” – **Male (IDM 015)** |
| Mediators  *Factors that enhancepre-existing internal behavioural values to enhance adherence to self-management practices* | Expectations | Wanting empathy | “If the doctor is kind, I will be more likely to agree with whatever is being told to me, because I feel like the doctor cares for me.” – **Male (IDM 012)**  “Doctors these days don’t even talk to you. They just look at you and prescribe the medicine…. If the doctor is nice, he will talk to them (patients) and that will help us improve the sickness as well” – **Female (IDM 003)**  “When patients come to see the doctor, we are already feeling down. So a few words of encouragement will go a long way.” – **Female (IDM 011)** |
|  |  | Wanting to reduce medications | “I hope that by reducing my sugar level, I can bring down the number of medicines.” – **Male (IDM 010)**  “The doctor keeps increasing the medication. I don’t like that feeling. I prefer it if I could reduce my medicine. At least then I won’t feel so sick.” – **Female (IDM 003)**  “I need to take so much of medicine. So many different types of medicine, every day… it’s killing me, doctor.” – **Female (IDM 021)** |
|  |  | Wanting doctors to interact better | “Of course, all advice given by the doctor is good, but there is a certain way to communicate that advice well.” – **Female (IDM 006)**  “If the doctor is nice to us and communicates well, it makes a huge difference. We will not stress out so much about being scolded for bad control. And we will be more open to volunteering information.” – **Male (IDM 023)**  “When a doctor speaks to a patient he should be gentle in the way he speaks. He shouldn’t get angry at us.” – **Female (IDM 022)** |
|  |  | Expecting same doctor to recall medical progress | “I feel it will be better if the doctor can remember the patient’s progress, so that the doctor can know if we’ve been taking the medications, whether to really increase or decrease the medicine.” – **Male (IDM 010)**  “It’s a lot easier if I get to see the same doctor, because he can easily know my progress, whether I am okay or not.” – **Female (IDM 021)**  “If it’s the same doctor, the doctor can know the patient better. I won’t even have to tell anything. They will already know.” – **Female (IDM 005)** |
|  |  | Wanting doctors to explain lab results trends | “I hope the doctor can update us better about our condition and give us a clearer picture on our health… once they get the results, I hope they can explain it to us clearly.” – **Male (IDM 002)**  “I will ask the doctor all these things, like blood sugar levels, cholesterol, urine, creatinine etc.” – **Female (IDM 011)**  “If the doctors are more approachable, I will ask them in detail the meaning of all my lab results.” – **Male (IDM 014)** |
|  |  | Requiring doctors to provide more information for their disease | “I feel its part of the doctor’s duty to educate the common person about the disease.” – **Male (IDM 014)**  “The only thing that I was told was that if I take my medicine, I’ll be able to control my disease. They never actually told me anything else about the disease. They have no time.” – **Male (IDM 010)**  “I feel that some times, the doctor doesn’t really give us any information.” – **Female (IDM 021)** |
|  |  | Motivating patient via feedback from doctor | “When I meet the doctor, and the doctor tells me to continue my medicine and that I’m getting well, then my moral will be high, my self esteem will be high.” – **Male (IDM 014)**  “When the doctor communicates well, I feel like I want to control my disease better.” – **Female (IDM 005)**  “If the doctor were to inform us, to properly tell us what areas we’re doing well, where we can improve etc. it will really motivate us.” – **Male (IDM 002)** |
|  | Health promotion | Receiving information about T2D management from doctor (diet,insulin, medication) | “So I asked my doctor about insulin, whether there are any side effects or anything. So then the doctor explained everything to me in detail.” – **Female (IDM 019)**  “I know about diabetes now. The doctors and the staff have educated me about this at length.” – **Female (IDM 024)**  “At that time I was very sad I had gotten diabetes. But the doctor advised me and motivated me. And I attended some talks given by the doctors.” – **Male (IDM 017)** |
|  |  | Discouraging against the use of CAM (doctor) | “…that’s when the doctor told me that these alternative medicines are not beneficial to my health.” – **Female (IDM 024)**  “We can’t be mad at the doctor if something goes wrong (after taking CAM). The doctor has already told us about CAM.” – **Female (IDM 006)**  “The doctor has already told me that they don’t recommend traditional medicine.” – **Male (IDM 004)** |
|  |  | Learning from DE about T2D management | “For example that time the diabetic educator and pharmacist gave us that talk on diabetes and medicine…” – **Male (IDM 018)**  “Then I met that diabetic educator, what’s her name?... ah yes, her. She told me about this disease and how to take my medication.” – **Male (IDM 004)**  “Then the diabetic educator told me how to take insulin correctly. And also she motivated me to live my life well.” – **Male (IDM 007)** |
|  |  | Receiving dietary advice from dietitian | “When you sit down with the dietician, yes they give you advice for your own good…” – **Male (IDM 012)**  “Previously when I was admitted, the dietician came and told me what foods to take, and how much.” – **Female (IDM 020)**  “Then I spoke to the dietician, and she told me what to eat, how I should eat more fruits and things like that. And so I did it for a year.” – **Female (IDM 024)** |
|  |  | Receiving medication advice from pharmacist | “There was this pharmacy person who instructed us how to take the medicine, and also how to store it.” – **Male (IDM 018)**  “I consulted the Alpro Pharmacist in Rasah Jaya.” – **Female (IDM 022)**  “So when I go to the pharmacy, I make it a point to ask about insulin. Then they’ll give me some information.” – **Female (IDM 019)** |
|  |  | Reinforcing insulin technique improves compliance | “When I was at the ward, they showed me how to correctly inject insulin.” – **Female (IDM 006)**  “When I was here, staff nurse A told me how to do it.” – **Female (IDM 020)**  “When I was hospitalized for my carbuncle, there they taught me how to inject my insulin.” – **Female (IDM 024)** |
|  | Support | Reminding to take medications (family) | “My wife helps me. She’s there to hold my shoulder. She helps remind me about medication.” – **Male (IDM 008)**  “My husband really takes care of me. He reminds me to take medicine.” – **Female (IDM 019)**  “Usually I control on my own, but if I forget to take medicine, and my reading goes high, she’ll start to nag me.” – **Male (IDM 017)** |
|  |  | Confessing family support is important | “Now both my children are working. But I still need them. I need their love and their support as I continue to take this medicine for the rest of my life.” – **Male (IDM 002)**  “Yes I believe family support is very important. I see this in my own family.” – **Female (IDM 011)**  “Family support is important. I support my wife in her diabetes…” – **Male (IDM 023)** |
|  |  | Knowing spouse is supportive | “Yes my wife is concerned about my health.” – **Male (IDM 002)**  “My wife is the person who told me that it’s better to take medicine from the hospital instead of all these traditional medicine.” – **Male (IDM 007)**  “My husband is very supportive. If I eat too much sugar, he’ll complain, and he also reminds me to take medicine.” – **Female (IDM 005)** |
|  |  | Reminding to control diet (family) | “My family doesn’t have much knowledge about diabetes. But my children sometimes remind me to control my diet.” – **Male (IDM 023)**  “My husband is very supportive. If I eat too much sugar, he’ll complain…” – **Female (IDM 005)**  “When I’m at home, my wife helps me maintain a strict control on my diet.” - |
|  |  | Sharing the burden of disease with family | “I talk to my family. I tell my wife about the disease and the medicine I take.” – **Male (IDM 004)**  “I talk to my mom about the disease. My mom is a diabetic too, and she’s also a nurse.” – **Female (IDM 021)**  “I don’t share my disease with other people, but my family, yes. My family knows about the disease.” – **Female (IDM 005)** |
|  |  | Knowing children are supportive and concerned | “My daughter is very supportive. She reminds me to take my insulin and even offers to inject me.” – **Male (IDM 012)**  “My children are all very supportive.” – **Male (IDM 007)**  “My children no longer live with me, but they still call up and ask if I’ve taken my medication.” – **Male (IDM 016)** |
|  |  | Knowing close friend who is supportive | “I have a friend, a colleague who always asks about my disease. He’s very concerned about me.” – **Male (IDM 002)**  “Now that I’ve been living here, I have some close friends who are always very concerned about my health.” – **Male (IDM 001)**  “I have a friend; we’ve been friends for 25 years. He’s very close to me. He always advises me about diabetes. He’s also a diabetic.” – **Male (IDM 013)** |
|  | Patient centeredness | Acknowledging doctors playing their role well | “Some doctors are really nice. They go the extra mile for you.” – **Male (IDM 008)**  “To me, sincerely, I think that what the doctors have done so far is really good. There is nothing more that I can expect out of them.” – **Male (IDM 017)**  “I don’t think that there are any problems with most doctors. They are only doing their duty.” – **Male (IDM 001)** |
|  |  | Acknowledging some doctors more approachable | “Some doctors take their time to explain things, and are very gentle.” – **Female (IDM 005)**  “Most doctors are very good, patient and friendly.” – **Female (IDM 011)**  “The doctor was willing to answer all my questions.” – **Female (IDM 021)** |
|  |  | Acknowledging interaction with doctors is cordial | “The relationship between my doctor and I is good.” – **Male (IDM 002)**  “I think my relationships with my doctors are fine.” – **Male (IDM 017)**  “I think I have an okay relationship with my doctor.” – **Female (IDM 024)** |
|  |  | Acknowledging HCP playing their role well | “The nurses also do their job well…” – **Female (IDM 003)**  “All the staff here are really efficient.” – **Female (IDM 021)**  “The service provided by the staff is very good. They are always smiling, willing to help.” – **Male (IDM 017)** |
|  |  | Preferring two way discussion with HCP | “If the doctor is willing to communicate well with you and show some concern, we patients would also be more forthcoming with our information.” – **Female (IDM 020)**  “The doctors should talk to the patients. Talk to us about our diet, our exercise. Don’t just order us to do things.” – **Female (IDM 005)**  “If the doctor is more conversant, I too will ask more questions and volunteer more information.” – **Female (IDM 006)** |
|  |  | Wanting doctors understand background & lifestyle | “It’s easy for the doctor to plan out how we should take our medication and eat. However implementing it isn’t so simple. Our lives aren’t as orderly as it’s made out to be.” – **Female (IDM 024)**  “The doctors don’t understand our lifestyle. They always interrogate us on why our sugar is high and accuse us of not doing certain things.” – **Female (IDM 005)**  “The doctor has his own duty to advise me. But I have a job as well, with its own struggles. And it’s not easy to just change things to suit the doctor.” – **Male (IDM 007)** |
|  |  | Showing concern for patients | “In my case, the doctors show a lot of concern.” – **Male (IDM 002)**  “In my opinion, the doctors are really striving to get you to lead a good life and be healthy.” – **Male (IDM 012)**  “I felt like the doctor looked after me like she was looking after her own son.” – **Male (IDM 007)** |
|  |  | Wanting doctors to spend quality time with patients | “The doctor needs to understand that although he sees 100 patients a day, the patient only sees 1 doctor in a few months. So the doctor needs to take time and talk to the patient.” – **Female (IDM 006)**  “There are some doctors that just copy the prescription and ask us to take it for the next 6 months. That’s not right. It would be more efficient if they spent more time talking to us.” – **Female (IDM 011)**  “They only focus on the HbA1c. They don’t really talk to you and ask you.” – **Male (IDM 010)** |
|  |  | Improving compliance by having empathy for patients | “If we feel cared for, as a patient, we would be more willing to take our medicine.” – **Female (IDM 003)**  “If the doctor talks to me nicely, smiles, cares for me, then I’ll be more receptive to the advice, and I’ll try to implement it. If the doctor calls me stubborn and scolds me, I’d just disregard everything that doctor says.” – **Male (IDM 015)**  “If the doctor communicates well, maybe the patient will also try to listen to what the doctor says and change their lifestyle.” – **Female (IDM 005)** |
|  |  | Negotiating the need to change dose with doctor | “I’ve tried to talk to the doctor to reduce the dose of my medicine…” – **Male (IDM 004)**  “I informed the doctor that I wanted to reduce my insulin dose. I asked her permission first. Finally we agreed on a value.” – **Male (IDM 014)**  “Every time the doctor increases the medicine dose, I will make it a point to ask the doctor for the reasons and the specifics.” – **Male (IDM 008)** |
