# Supplementary material for: The external realities of people with type 2 diabetes—Understanding disease perspective and self-management behaviour via Grounded Theory Approach
Source: PLoS One. 2021 Jan 14;16(1):e0245041. doi: 10.1371/journal.pone.0245041 (PMC7808602; doi:10.1371/journal.pone.0245041)
Supplement: Attachment — Submitted filename: PLOS ONE Swarna Nantha - External Reality (21.2.2020).docx [file pone.0245041.s006.docx]

**The External Realities of People with Type 2 Diabetes – Understanding Disease Perspective and Self-Management via Grounded Theory Approach**

Yogarabindranath Swarna Nantha^a,b,c*^

Azriel Abisheg Paul Chelliah^a,b^

Shamsul Haque^c^

Gan Kim Yen^a,b^

^a^Primary Care Research Initiative And Methods Education Department (PRIMED), Seremban Primary Health Care Clinic, Jalan Rasah, 70300 Seremban, ^b^Non-Communicable Disease Department, Seremban Primary Health Care Clinic, Jalan Rasah, 70300 Seremban, Malaysia, ^c^Department of Psychology, Jeffrey Cheah School of Medicine and Health Sciences, Monash University Malaysia

^*^Corresponding Author: Dr. Y. Swarna Nantha, Primary Care Research Initiative And Methods Education Department (PRIMED), Seremban Primary Health Care Clinic, Jalan Rasah, 70300 Seremban, Malaysia, E-mail: yogarabin@gmail.com

**ABSTRACT**

**Background**

Qualitative strategies can uncover the relationship between the external realities of people with type 2 diabetes (T2D) and obstacles to disease self-management. In-depth interviews and focus group discussions (FGD) could be used to devise psychological models that can facilitate behavior changes in people with T2D. We aim to identify salient factors that govern the immediate realities of people with T2D in relation to disease management.

**Methods**

A qualitative study was conducted at a regional primary care clinic in Malaysia using a Grounded Theory Approach. Participants with T2D were recruited through purposive sampling in a bid to determine their living experiences with the disease. A total of 34 in-depth interviews with 24 people with T2D and 10 health care professionals, and two FGDs with people who have T2D were conducted.

**Results**

Participants’ self-management practices and disease perspectives are arbitrated by three major processes – 1) external reality, 2) internal reality, 3) mediators of behaviour. Within the context of external reality, three important sub-themes were identified – fixed background status, personal experience, and world view. Lifestyle habits of persons with T2D play an important role in disease management. Issues about poor food environment in the country appear to be a common recurring concern. Participants have a high degree of expectations for a more person-centered approach to their illness.

**Conclusions**

We identified modifiable and non-modifiable behavioural factors that influence the daily living environment of participants with T2D. This information can be used to customize the management of T2D through targeted behavioural interventions.

**KEYWORDS:** Type 2 Diabetes; self-management; engagement; Grounded Theory; Non-communicable diseases.

**Introduction**

Malaysia has witnessed an 80% increase in the prevalence of diabetes in a span of just a decade[1]. The numbers continue to grow as the prevalence of type 2 diabetes (T2D) saw a sharp increase from 11.6% in 2011 to 17.5% in 2015[2]. Unexpectedly, the availability of widely accessible public primary care clinics do not appear to translate into effective glycemic management in people with T2D [3]. Instead, the country experienced a stagnation in the overall glucose levels of people with T2D with mean HbA1c levels remaining at 8.2% between the year 2009 and 2012[4]. The reasons for this predicament are scarcely explained in literature. Experts allude to lapses in medication taking, food choices and the lack of physical activity as the primary reason for these trends[3]. Therefore, a focused qualitative inquiry in this direction is necessary to identify pitfalls in disease self-management.

Effective implementation of self-management practices (engagement in treatment plan, dietary measures, and exercise) have the potential to generate increased glycemic stability in people with T2D [5]. However, T2D self-management practices rely heavily on the habits that permeate the daily life of an individual. Self-management requires changes in the pre-existing health belief and behaviour seen amongst T2D patients within the context of their ‘living environment’. This obstacle remains as the single most difficult challenge in T2D disease management[6–8]. Hence,there is a need for a qualitative exploration of the practicality of broadly accepted self-management practices that are routinely prescribed for T2D patients.

In this study, we aim to develop a quantifiable theoretical model that could explain disease management in T2D patients[9]. We intend to explicate the salient factors that dictate the naturalistic environment of T2D patients through a Grounded Theory Approach. In this manuscript, we will present a conceptual framework derived from the results of the current study by discussing extensively about the ‘external reality’ of the person with diabetes.

**Material and methods**

**Design**

A qualitative grounded theory approach was chosen in order to identify behavioural patterns relevant to the objectives of this study. An inductive approach to qualitative inquiry was utilized to 1) assist the discovery of ‘real-world’ or ‘naturalistic’ themes, 2) ensure that codes remain closely grounded to emergent data, and 3) avoid a simplistic assessment by obviating the utilization of antecedent behavioural theories seen in literature[10]. This study protocol received approval from the Medical Research and Ethics Committee, Ministry of Health Malaysia (NMRR-18-151-39886) on the 30th of May 2018 and also Monash University Human Research Ethics Committee on 18^th^ of October 2018 (Project ID: 17062). All subjects in the study gave informed consent prior to participation.’

**Participants and Setting**

A purposive sample was recruited from a dedicated NCD department at the Seremban Primary Care centre in Malaysia, a large-scale general practice clinic within the district of Seremban. Maximum sampling strategy was employed to achieve a good spread in terms of socioeconomic and disease control indicators (Table 1 and Table 2). A published study protocol of this endeavour provides a more detailed description of the research methods employed here[9].

Table 1. Demographic details of T2D patients of the study

| **Subject Characteristics** | ***N*** |
| --- | --- |
|  |  |
| **Gender** |  |
| Male | 15 |
| Female | 9 |
|  |  |
| **Age In Years** |  |
| < 30 | 1 |
| 31 – 50 | 6 |
| 51 – 60 | 7 |
| 61 – 70 | 7 |
| > 70 | 3 |
|  |  |
| **Ethnicity** |  |
| Malay | 12 |
| Chinese | 1 |
| Indian | 10 |
| Others | 1 |
|  |  |
| **Marital Status** |  |
| Unmarried | 3 |
| Married | 19 |
| Divorced | 1 |
| Widow/Widower | 1 |
|  |  |
| **Work Status** |  |
| Not working | 12 |
| Working Full-time | 8 |
| Working Part-time | 1 |
| Self-employed | 3 |
|  |  |
| **Level Of Education** |  |
| Certificate level | 19 |
| Diploma | 3 |
| Bachelor’s degree | 2 |
|  |  |
| **Diabetes duration (years)** |  |
| < 5 | 5 |
| 6 – 10 | 3 |
| 11 – 15 | 4 |
| 16 – 20 | 2 |
| > 21 | 3 |
|  |  |
|  |  |
|  |  |
| **Number of medications** |  |
| 3 – 5 | 12 |
| > 6 | 12 |
|  |  |
| **Type of medication** |  |
| Oral only | 5 |
| Insulin only | 4 |
| Combination | 15 |
|  |  |
| **Glycaemic status** |  |
| 6.5 – 8.0% | 3 |
| 8.1 – 9.0% | 9 |
| 9.1 – 10% | 4 |
| > 10% | 8 |
|  |  |
| **Complications** |  |
| No | 17 |
| Yes | 7 |
|  |  |
| **Number of comorbidities** |  |
| 1 | 13 |
| 2 | 4 |
| > 3 | 8 |
|  |  |

**Table 2. Demographic details of healthcare professionals involved in the study**

| **Subject Characteristics** | ***N*** |
| --- | --- |
|  |  |
| **Profession** |  |
| Diabetes educator (nurse) | 5 |
| GP | 3 |
| Pharmacist | 2 |
|  |  |
| **Mean Age In Years** | 34 |
|  |  |
| **Mean Years In Service** | 10 |
|  |  |
| **Ethnicity** |  |
| Malay | 7 |
| Chinese | 1 |
| Indian | 2 |
|  |  |
| **Marital Status** |  |
| Unmarried | 2 |
| Married | 7 |
| Divorced | 1 |
|  |  |
| **Level Of Education** |  |
| Diploma | 3 |
| Bachelor’s degree | 5 |
| Master’s degree | 2 |
|  |  |

**Data Collection**

All 34 in-depth (24 patients and 10 health care professionals) interviews and 2 focus group discussions (comprising 8 patients each session) took place between May 2018 to April 2019. Participants were interviewed in a designated research room within the premises of the clinic. A previously published protocol explains details related to interviewing technique and the topic guide that was employed in this study[9]. We minimized the framing of questions based on the pre-conceived notion of the interviewer by allowing participants to express issues perceived salient to them. No new themes or categories were obtained after the 10^th^ round of interviews and theoretical saturation was considered to have been achieved. However, the establishment of the validity of these categories required additional interviews with 14 participants where these narratives were further explored, enriched, and consolidated.The interviews were audiotaped and transcribed verbatim. One researcher (YSN) conducted all interviews.

**Data Management and Analysis**

Once data collection was complete, individual transcripts were 1) proofread independently, 2) re-examined by the investigator, and 3) cross compared using iterative methods. Proofread transcripts were then entered into a qualitative software (Atlas.TI qualitative analysis program, Version 7, Cincorn Systems Inc, 2008) and coded manually to identify specific themes by YSN. The data was coded in accordance with the classical and constructivist Grounded Theory methods [10,11].

The coding of data involved a multi-stage process. First, all the codes seen in the transcripts were contrasted with each other to identify emerging themes.Then, the responses of the participants from the qualitative study were analyzed by the first author (YSN). This step was followed by a scheduled discussion with another author (SH) to categorize the data according to relevant emerging themes. This method of constant comparison was carried out between researchers throughout the entire process of the study to refine the acquired codes within the transcript. Transcripts were finally coded independently by two coders and a list of emerging themes and categories were generated. Excellent inter-coder consistency was found (Cohen’s κ =0.84). Also, themes derived from this study were assessed by a panel of experts and showed excellent content consistency (Cohen’s κ =0.88).

**Trustworthiness**

Trustworthiness of this study was assured by using different techniques of data collection through both in-depth interviews (IDI) and FGDs (methodological triangulation)[12]. Two researchers performed data analysis and interpretation (investigator triangulation). We chose to validate the information (data triangulation and negative case analysis) by comparing interviews that were obtained from diverse sources (people living with T2D and healthcare providers). Additionally, the authors interpreted, conceptualized and revised behavioural constructs at various stages of the study via an iterative process (persistent observation).

**Results**

The findings identified participants’ general views, thoughts, perceptions and actions about their behaviour towards disease management. The analysis of participants’ description of their self-management practices and disease perspectives resulted in the generation of 3 main categories – 1) external reality (the physical environment), 2) internal reality (the inner psychological environment and personal volition), and 3) mediators of behaviour (Figure 1). Furthermore, the narratives from FGDs (people with T2D) and IDIs (healthcare providers) helped consolidate the linkages between observed thought patterns on the basis of similarity of context. This verification laid the foundation for the creation of a theoretical model consisting of 7 broad themes (Figure 1). The constructs subsumed by these broad themes occur simultaneously and in an interactive manner. In this manuscript, we will be discussing the themes forming the ‘external reality’ of the framework - fixed background status, (ii) personal experience, and (iii) world view (Table 3).

**Figure 1: Conceptual framework describing the external reality, internal reality and mediators related to self-management of T2D**

Table 3: Categories, definitions, frequency, and codes describing the external reality of T2D patients

| **Category** | **Sub-Category** | **Frequency** | **Codes** |
| --- | --- | --- | --- |
| **Fixed background status**  Non-modifiable factors that shapes the daily lives of the patient | Lifestyle  Sociodemographics  Food Environment | 212  135  44 | Inconsistent medication taking (or medication taking behaviors)  Timing of medications  Practicality of prescribed advice  The influence of age  The influence of education  Cultural paradigm  Affordability  Needing to eating out  Poor food quality  Solutions to existing milieu |
| **Personal experience**  Physical and emotional experience pertinent to the phenomenon of living with diabetes | Experiential  Adverse  Exemplar  Uncertainty  Wellbeing  Influence  Relationship with GPs  Service | 196  156  154  137  90  74  49  44 | Experiencing physical symptoms  Response of body to medications  Experiencing side effects  Coping with side effects  Witnessing complications  Emulating exemplary patients  Becoming role models  Nature of disease  Unpredictability of disease despite compliance  Positive effects of using modern medications  Positive effects of using alternative medications  Influence of family members  Influence of friends  Positive disposition of doctors  Negative disposition of doctors  Opinion about services  Opinion about doctors |
| **World view**  Self and society’s point of view about disease | Health Belief  Knowledge  Perception | 145  125  74 | Beliefs about complementary alternative medications  Beliefs about medication, illness, diet and exercise  Mechanism for T2D  Recognizing complications  Perception of self  Perception of others and society  Devolving autonomy doctors |

**Fixed background status**

**Lifestyle**

In general, people frequently miss taking their medication when they are out of their homes. They often don’t bring along their medications and cite forgetfulness as their main shortcomings. Similarly, people leading hectic daily routines (constantly being in a rush, or immersed in completing daily chores) also tend to take their medications inconsistently.

Most patients (as do pharmacists) concur that inherent lifestyle habits, especially work, contribute to the difficulty of taking medication at the recommended time. They also feel taking oral medications is much more convenient compared to insulin, especially when they are on the go.

“Maybe insulin yes, I do agree because they will need to store it somewhere and excuse themselves in order to find a suitable place to inject it. But tablets are not a problem at all. When you are sitting at the restaurant while eating, you readily have it in your pocket. After having lunch, you just place it in your mouth and drink a glass of water. That’s it” (IDM 011)

Patients also find it hard to engage in therapeutic lifestyle changes such as dietary measures and exercise. They feel that the prescribed dietary advice is often impractical. Most importantly, they blame work constraints as a major reason for food choices. Maintaining exercise becomes a difficult habit to sustain as they are overwhelmed with work responsibilities and household chores.

“Sometimes when you come home from work, you don’t feel like doing exercise, especially when you come home late. You ask yourself ‘Why must I go out and do exercise?’, I feel my household chores are already a hand full” (IDM 005)

**The dynamics of sociodemographic status, cultural paradigm and cost impediments**

Most patients dismiss the relevance of age in relation to their commitment to stringent disease management. They say that “there is no difference; it all depends on the willingness to follow the doctor’s advice”. Nevertheless, participants agree some individuals tend to ignore the consequences of their disease when they are young but become increasingly concerned about it later in life.

Similarly, patients feel the level of education is not a measure of eagerness to manage their disease. But participants do concede that people with lower education background are more likely to rely on the advice of their doctor. They also reckon that more educated patients are well placed to make their own decisions about their disease management.

“Look, even if you hold a master’s degree, there is no guarantee you will look after your own wellbeing. I think there is no relationship between being a responsible patient and owning a degree. No I don’t think so” (IDM 015)

From a cultural standpoint, Muslim patients express having doubts over the “kosher status” of insulin. Some also feel that taking insulin prevents them from performing religious fasting practices out of the fear of hypoglycemia. From a financial standpoint, many patients cite affordability as a reason for inconsistent self blood glucose monitoring at home. They claim the cost of purchasing glucose strips can be very expensive.

“The glucometer strip costs a small fortune you know. 39 ringgit for just 25 strips” (IDM008)

**Food environment**

During FGDs, most patients appear to have a negative perception of the quality of meals served at food outlets. One patient blamed “unscrupulous food vendors for adding too much sugar in their cooked meals”. Nevertheless, they are compelled to dine out due to the nature of their work and the size of the family unit. They also question the lack of dietary options to choose from that best suit their disease. As a result, the majority of patients have the tendency to violate dietary advice (at times seem to condone this “infrequent behaviour”) while eating out. To curb this problem, both GPs and patients have suggested “the need for legislative action” designed to uphold food standards in the country. From a more personal perspective, patients also strongly feel regularly consuming meals at home can help them to meet their dietary goals.

“My wife says she will help prepare a much healthier diet while I am at home. So we ensure we take food which contains more fibre and lesser amounts of sugar. I avoid sugary beverages if I can. I try my levels best not to consume when I am out of the house because it’s a healthier eating at home. We eat more vegetables.” (IDM 014)

**Personal experience**

**Adverse effects**

In general, patients commonly experience very little side effects with their medications. When it does occur, patients cite physical fatigue as the commonest medication adverse effect, followed by dizziness, hypoglycemia, gastrointestinal problems and loss of productivity. Any noticeable reaction to medication also leads to self-alteration of the recommended dosage.

“The doctor told me to take two tablets right? But I get really feel lethargic and dizzy when I take 2 tablets. So I reduced it to just 1 tablet myself” (IDM 007)

**Experiential reflections**

Patients feel their bodies respond differently to medications, claiming that sometimes “it takes a longer time for medications to be effective”. This subjectivity varies from one individual to another.

“Based on my experience, previous drugs didn’t really work for me. Only this current drug, I could see changes within the week.” (IDM 004)

“It may vary from person to person. Some may react differently. For me, I know, 2 tablets is the best for my body.” (IDM 012)

In a similar vein, some patients with poorly controlled diabetes have a higher degree of sensitivity to the specific physical manifestation (lethargy, frequent micturation, hypoglycaemia or body aches) while some patients remain unaware. These symptoms appear to be more prevalent when they “keep missing medications [deliberately] for a prolonged period of time”.

**Uncertainty and wellbeing**

It is not uncommon for some participants to exhibit elements of uncertainty and frustration in managing their disease. They invoke feelings of “I feel alright today, but I am not sure about tomorrow” despite all attempts to effectively handle their disease.

“I know it is important to control my disease. I just feel afraid of the disease sometimes. I am just troubled by the fact that anything [complications] can occur at anytime [beyond control].” (IDM 006)

However, they gain more confidence by observing improved physical wellbeing as a result of consuming either modern or complementary alternative medication (CAM).

“I feel much better right now [after taking medications]. I feel physically healthy and my work performance is much better. I no longer feel lethargic.” (IDM 007)

“I have not been compliant with my medications you know and my blood glucose readings [glucometer] were at 9 or 10. But then once I consume the blended bitter gourd juice, I realize the readings tend to normalize.” (IDM 10)

**Exemplar and the influence of others**

The motivation for a more disciplined approach to T2D management arises from witnessing the consequence of complications in other patients. This direct observation triggers a certain level of openness and participants become more receptive towards learning from people who manage their diabetes effectively.

“I have a friend who called me up saying, ‘I am doing fine without medications’. A month later, I receive a call from that same friend saying in a distressed voice that he recently had an episode of stroke. So medication is something that I must take accordingly” (IDM 017)

They eventually adopt the position of ‘role models’ to both friends and family members. On the other hand, some acknowledge receiving incorrect advice from some T2D patients or even family members. Although a positive relationship among family members supports disease management in most cases, some family members unduly influence patients to sidestep disease management by avoiding medications.

“I have heard people saying taking too much tablets can kill you at the end of the day” (IDM 005)

“My family members, especially my sister, she keeps telling me to avoid insulin and get a second opinion instead. When she knows I am going to meet a doctor, she insists that I tell them I am not willing to use insulin” (IDM 020)

**Opinion about primary care services**

Patients are largely satisfied with the way the primary care clinics operate, stating that the quality of medications and clinical consultations are excellent.

“The services provided here is excellent. I have no issues at all.” (IDM 017)

“I will say it (the service provided) is good. Everyone (healthcare providers) really do care and help the patient recover.” (IDM 013)

“The consultation is good, and they (healthcare providers) do what is really necessary.” (IDM 001)

However, they claim seeing different GPs during each consultation often leaves them confused as they receive conflicting opinions about disease management. Participants were also disgruntled with the way GPs pay very little attention to their needs because they see too many patients in a day. In the same way, several nurses recalled patients complaining that “consultations with the GP were so swift” and doctors “barely had time to address any of my questions”.

“Probably because they (GPs) are stressed when they see too many patients. When you see the doctor, he just tells you what your current medical status is like and prescribes you medications. That’s about all.” (IDM 002)

“Maybe he (doctor) is too busy with many patients, he doesn’t have the time to explain anything to me.” (IDM 011)

“They stare at you, look at your results, and prescribe the same old medications for the next four months. But I don’t blame them, they probably have no time.” (IDM 010)

“They (GPs) just prescribe the medication, and off I go. Some of them don’t even talk to you.” (IDM 003)

**Relationship with GPs**

Patient’s interaction with GPs depends on “which type of doctor” they see on the day of the consultation. In general, patients perceive their GPs as agreeable during consultations and consider them as “health advisors”. Nevertheless, patients sense unpredictability in the demeanour of GPs, ranging from being “relatively quiet” to being “very approachable”. Large number of patients were disappointed with the attitude of some GPs who appear to be lacking “warmth” or empathy. Small numbers of GPs are noticeably rude, condescending or even disparaging towards patients.

“Some doctors are nice. They go the extra mile. But some doctors just give you the cold shoulder treatment.” (IDM 008)

“There are doctors who threaten us. They say if you don’t take your medication, you might end up dying faster” (IDM 003)

Missing narrative on “Services” sub theme…

**World view**

**Perception about disease**

Participants perceive that diabetes is “becoming a norm” in the society and are aware about the seriousness of the disease in the country. Health care professionals substantiate this notion by stating that patients often adopt the mentality of “everybody has diabetes nowadays”. The majority of T2D patients feel that they have poor control over their disease. Only a fraction of patients are confident and declare they have optimal disease control. A certain degree of self-empowerment seems to be lacking insofaras participants place absolute confidence in the authority of the doctor.

“Yes. Common. Diabetes is very common in our society.” (IDM 011)

“I don’t know whether the medication really helps me or not. I wouldn’t know. Because every time I come to the clinic, the doctor looks at me and says ‘you have to take this medicine’. So, when you have to take medicine, you just take it. I don’t do much research about these things” (IDM 010)

**Health Beliefs**

Patient’s opinion about their disease is dominated by several salient health beliefs. For example, patients feel that T2D can reduce their quality of life as it is closely linked to other illnesses such as hypertension and coronary heart disease. Many participants believe the way glucose is metabolized in the body varies from one individual to another.

There is a prevailing thought that taking too much medication can cause ill health. “Medication can cause kidney damage” is a recurring concern expressed by almost all patients. This detail was further corroborated by interviews with GPs as well. Patients also feel insulin has many side effects and equate the commencement of insulin as having reached a “critical stage of the disease”.

“People consider us as having severely uncontrolled diabetes when we mention that we have started taking insulin.” (IDM 018)

“It’s the perception people have you know. Once you start taking insulin, they think that our disease is out of control and that anything can happen to us. It’s just a matter of time.” (IDM 004)

A small group of patients believe T2D can be managed via lifestyle modifications alone. This misconception fuels the need to consume rice sparingly. They also consider the idea of “cooking, sweeping, cleaning and washing” as an equivalent to exercise.

“I think even if you stopped taking any medicine, I am certain you can bring down your sugar levels - you just need to do exercise regularly and have restraint over your food intake. If you are consistent, I believe you can bring down your sugar levels.” (IDM 010)

“I sweep, cook, and do the laundry everyday. I mean when I walk upstairs and down, I feel that is a form of exercise. I sweep the floor and clean the house. And I sweat it out too you know. I don’t know it’s true or not, but I think that’s enough for me.” (IDM 005)

**Knowledge**

Patients commonly associate the cause for T2D with old age, genetic predisposition, deficiency of insulin and a non-functioning pancreas. They also recognize important complications of T2D such as kidney damage, coronary artery disease, and neurological disorders.

“I know diabetes runs in the family. If your dad has it, your mom has it, then 95% of the time you end up getting it too.” (IDM 008)

“I have this fear that diabetes will lead to kidney problems in the future. It will severely limit my daily activities as I might to undergo hemodialysis when that happens.” (IDM 021)

**Discussion**

**Summary of main findings**

We uncovered an intricate web of interaction determining the dynamics of self-management practices in T2D patients. The psyche of the conventional patient is exemplified by a rich interplay between their external reality, inner psychological environment and appropriate behavioural mediators (Figure 1). Thus, this framework helps 1) summarize this behavioural complexity within an operational platform and 2) demarcate clear margins amenable to behavioural intervention or preventive measures.

In this article, we summarize the thoughts and beliefs in relation to the external realities that shape the daily lives of an archetypal T2D patient. Three major themes were identified: fixed background status, personal experience, and world view. Sub-categories within each theme can be further classified as modifiable (lifestyle, health belief system, general perception, knowledge, patient-doctor relationship, health services) and non-modifiable (sociodemographic status, the influence of others, food environment) behavioural factors (Appendix A, B and C).

Need more discussion. Tie this all together. It’s a LOT of information (findings) and yet how can I use this in practice?

**Strengths and Limitations**

This study was conducted with participants originating from only one regional primary care institution. However, the richness and the quality of the data was established through the procees of triangulation where selected healthcare professionals from adjacent clinics were interviewed to verify the views expressed by the participants. Additionally, we emphasized on inclusivity by obtaining participants 1) from a community-based setting and 2) with a mixed sociodemographic background. Qualitative results cannot be generalized…

**Comparison with literature**

The optimal metabolic control of T2D involves a multifactorial process requiring a focused assessment of different facets of the living environment of the patient[13]. Our study demonstrates that the motivation behind self-management practices in T2D patients is closely linked to the understanding of the dynamics of their lifestyle habits. GPs play an important role in helping patients overcome their volitional inertia and to follow through on proper initiatives that facilitate behavioural change. A person-centered approach could ameliorate this situation by encouraging GPs to pay more attention to the background status and expectations of the patients[13].

Our study highlights the paternalistic doctor-patient communication style prevalent in most Southeast Asian countries[14]. Similarly, health services are overwhelmed by high patient load which severely limits constructive consultation time[14]. This situation in part gives rise to patients’account of disagreements with GPs described in the form of 1) having received insufficient advice and 2) GPs reportedly being poor mannered or inattentive[15,16]. Hence, a time-constrained working environment prevents a more participatory role of consultation, which in turn, hampers empathy towards patients.

The poor conditions and the lack of access to healthier options within the immediate food environment appear to be a constant pre-occupation amongst participants in this study. The results of this study suggest that exposure to ‘hyperpalatable food’ products is associated with food craving tendencies in T2D patients[17]. This phenomenon is evidenced by breaches in dietary adherence despite being fully aware of the consequences of committing such violations. Strikingly, an offshoot of this larger study discovered a close association between both food addiction scores and binge eating severity with the diabetes status of the patient[18].

**Implications for future research and clinical implications**

The categories described in this study serve as potential opportunities to help people with T2D make behaviour changes by addressing their commonly held doubts and fears.However, these variables only serve as a starting point for targeted interventions - other methods should be employed in conjunction with these findings to increase the likelihood of behaviour change in T2D patients. One plausible technique would be the use of persuasive communication strategies to help create a more empathetic environment for both patients and GPs[6]. Negative perceptions and adverse disease experience can also be mitigated by conducting T2D self-management group sessions by popular opinion leaders (i.e. exemplary patients and expert physicians) using interpretations from real-world parameters depicted in this study[6].

**Conclusion**

The results from this study are important for healthcare providers as it underscores common self-management pitfalls faced by T2D patients in their interaction with the healthcare system and their immediate social surrounding. This information can be used to customize the management of their disease by conducting targeted behavioural interventions in accordance with their external realities.

**Acknowledgements**

We would like to thank Monash University of Malaysia for granting us theopportunity and technical support requisite to carrying out this project. The main idea for the conceptual framework in this study was conceived from an epiphany I experienced while on a plane ride from Australia to Malaysia,	due in large part to Mr. Rodney Smith’s seminal work called ‘Awakening: A Paradigm Shift of the Heart’. Last but not least, we would like to express our heartfelt gratitude all T2D participants of this study, who were in all practical sense, the ‘true authors’ of this manuscript.

**Conflict of Interest**

The authors declared no potential conflicts of interest with respect to the research, authorship, and/or publication of this article.

**References**

1.	Letchuman GR, Wan Nazaimoon WM, Wan Mohamad WB, et al. Prevalence of diabetes in the Malaysian National Health Morbidity Survey III 2006. Med J Malaysia. 2010;65: 173–179.

2.	Institute for Public Health. National Health and Morbidity Survey 2015 (NHMS 2015). Vol. II: Non-Communicable Diseases, Risk Factors & Other Health Problems. Putrajaya; 2015.

3.	Hussein Z, Taher SW, Gilcharan Singh HK, Chee Siew Swee W. Diabetes Care in Malaysia: Problems, New Models, and Solutions. Ann Glob Heal. 2015;81: 851–862. doi:10.1016/j.aogh.2015.12.016

4.	Non-Communicable Control Division. National Diabetes Registy Report. Putrajaya; 2012.

5.	Shojania KG, Ranji SR, McDonald KM, et al. Effects of Quality Improvement Strategies for Type 2 Diabetes on Glycemic Control. JAMA. 2019;296: 427–440.

6.	Glanz K, Rimer K, Viswanath K. Health Behaviour: Theory, Research and Practice. 5th ed. San Francisco: Jossey-Bass; 2015.

7.	Tricco AC, Ivers NM, Grimshaw JM, Moher D, Turner L, Galipeau J, et al. Eff ectiveness of quality improvement strategies on the management of diabetes : a systematic review and meta-analysis. Lancet. 2012;379: 2252–2261. doi:10.1016/S0140-6736(12)60480-2

8.	Narayan KM, Ali MK, Koplan JP. Global Noncommunicable Diseases — Where Worlds Meet. N Engl J Med. 2010;363: 1196–1198.

9.	Swarna Nantha Y, Haque S, Swarna Nantha H. The development of an integrated behavioural model of patient compliance with diabetes medication : a mixed-method study protocol. Fam Pr. 2018; 1–6. doi:10.1093/fampra/cmy119

10.	Charmaz, K. Constructing Grounded Theory - A Practical Guide through Qualitative Analysis. 1st ed. London: SAGE Publications Ltd.; 2006.

11.	Corbin J, Strauss A. Basics of qualitative research: Techniques and procedures for developing grounded theory. 4th ed. California: SAGE; 2015.

12.	Brien BCO, Harris IB, Beckman TJ, Reed DA, Cook DA. Standards for Reporting Qualitative Research : 2014;89: 1245–1251. doi:10.1097/ACM.0000000000000388

13.	Shrivastava SR, Shrivastava PS, Ramasamy J. Role of self-care in management of diabetes mellitus. J Diabetes Metabolic Disord. 2013;12: 14.

14.	Claramita M, Utarini A, Soebono H, Van Dalen J, Van Der Vleuten C. Doctor-patient communican in a Southeast Asian setting: the conflict between ideal and reality. Adv Heal Sci Educ. 2011;16: 69–80.

15.	Annandale E, Hunt K. Accounts of diagreements with doctors. Soc Sci Med. 1998;46: 119–129.

16.	Gandhi IG, Parle JV, Greenfield SM, Gould S. A qualitative investigation into why patients change their GPs. Fam Pract. 1997;14: 49–57.

17.	Gearhardt AN, Grilo CM, DiLeone RJ, Brownell KD, Potenza MN. Can food be addictive? Public health and policy implications. Addiction. 2011;106: 1208–12. doi:10.1111/j.1360-0443.2010.03301.x

18.	Swarna Nantha Y, Kalasivan A, Ponnusamy Pillai M, et al. The Validation Of The Malay Yale Food Addiction Scale 2.0: Factor Structure, Item Analysis and Model Fit. Public Health Nutr. 2019; 20: 1 - 8
