# Supplementary material for: The external realities of people with type 2 diabetes—Understanding disease perspective and self-management behaviour via Grounded Theory Approach
Source: PLoS One. 2021 Jan 14;16(1):e0245041. doi: 10.1371/journal.pone.0245041 (PMC7808602; doi:10.1371/journal.pone.0245041)
Supplement: Attachment — Submitted filename: Response To Reviewers (21.10.2020).docx [file pone.0245041.s007.docx]

**Response to Reviewers**

*Comments from Academic Editor*

1. Please include additional information regarding the interview guide or script used in the study and ensure that you have provided sufficient details that others could replicate the analyses. For instance, if you developed a guide as part of this study and it is not under a copyright more restrictive than CC-BY, please include a copy, in both the original language and English, as Supporting Information

- Thank you for this comment. We have included this our topic guide (for IDIs and FDGs) in the Supporting Information 1 document. We have also made revisions to manuscript to reflect these changes:

Page 7, Paragraph 1, Line 6 - 7

A previously published protocol explains details related to interviewing technique and the topic guide (Supporting Information 1) that was employed in this study[12].

2. Please amend either the title on the online submission form (via Edit Submission) or the title in the manuscript so that they are identical

- Thanks for pointing out this discrepancy. We have edited the manuscript to ensure consistency.

3. Please include captions for your Supporting Information files at the end of your manuscript, and update any in-text citations to match accordingly

- These recommended changes have been made to the manuscript

*Comments from Reviewer 1*

1. The language throughout the paper is judgmental and needs to be revised using strengths-based, person-centered, and empowering messages. Negative, judgmental language is not only unhelpful to people with diabetes and health care providers, it negates much of what you found in your study. Please see my notes in the attached word document, where I have gone through the entire manuscript, made suggestions, and highlighted concerning examples. Please refer to the guidance published by the American Diabetes Association and Association of Diabetes Care and Education Specialists: Dickinson, et al. (2017). The use of language in diabetes care and education. Diabetes Care, 40, 1790-1799, which includes recommendations for changing specific

words/phrases and rationale for doing so.

- We would like to express our gratitude for investing the time to look through the entire manuscript and providing us an annotated version of the manuscript to ease the task of revision. We have examine the suggested guidelines and tried our best to incorporate these elements to our article.

2. Only 7 of 18 references represent current literature and a few references are extremely dated.

- We have removed dated references and included articles that were published primarily between years 2010 – 2020.

3. I’m not sure what a “quantifiable theoretical model” is. How can you quantify results of a grounded theory approach? Clarify or remove the word “quantifiable”

- We agree with this comment and made appropriate changes to the manuscript:

Page 2, Paragraph 3, Line 24 - 25

This study builds upon a larger initiative aimed at developing a comprehensive qualitative framework that could help explain disease management in people with T2D[9, 10].

4. The Conceptual Model depicted in Figure 1 is not quantitative and it is labeled “conceptual” and not “theoretical.” These terms get used interchangeable, though some define them separately. If you are referring to the same thing here, be consistent in terminology.

- Thanks for this comment. We have made changes to maintain consistency in terms of terminology used throughout the manuscript.

5. Can you reword this sentence to something that is more clear? I’m not really sure what you mean by this 🡪 avoid a simplistic assessment by obviating the utilization of antecedent behavioural theories seen in literature

- We concur with this comment. We have made changes to simply the sentences to better reflect what we had intended to convey to the reader:

Page 4, Paragraph 2, Line 15 - 16

*remain open* to newer insights instead of forcing data to fit antecedent behavioural concepts seen in literature[11, 12]

6. Spell out the first time and put abbreviation in parentheses.

We have revised this accordingly:

Page 4, Paragraph 3, Line 23 - 24

A purposive sample was recruited from a dedicated non-communicable disease (NCD)department…

7. What did this strategy consist of (maximum sampling strategy)?

Thanks for this comment. We have added an additional statement to clarify this terminology:

Page 4, Paragraph 3, Line 26 - 27

This step involves the purposeful selection of cases to document diversity and elicit common behavioural patterns related to dimensions of interest[12].

8. It is not clear to me how this minimized pre-conceived notions of the interviewer…

- Thank you for this comment. We have edited this sentence to bring about more clarity in what was intended to be conveyed to the reader:

Page 4, Paragraph 3, Line 26 - 27

To address the limitations of positionality, we minimized the framing of questions based on the pre-conceived notion of the interviewer by i) taking up the role of a neutral observer, ii) remaining as far as possible an outside expert during interviews, and iii) allowing participants to freely express issues perceived salient to them[15]

9. I found this confusing and had to spend a lot of time going back to the abstract, intro, etc., to figure out why only this section was being discussed. Is it possible to more clearly explain this up front? Why did you focus on this section only. After a lot of thought I came up with some ideas and maybe other readers would too; just thinking you could speed up the process (connection) for the reader.

This observation is correct. We have made the necessary changes to the introductory section and results section:

Page 3, Paragraph 3, Line 22 - 29:

This study builds upon a larger initiative aimed at developing a comprehensive qualitative theoretical model that could help explain disease management in people with T2D[9, 10]. In previous papers, we have expounded on the drivers of 'internal realities' related to self-management behaviour namely, 1) specific mediators, and 2) observed performance self-care activities[9, 10]. In the current article, we intend to expound on the immediate physical and sociocultural that dictate the naturalistic environment of T2D patients through a Grounded Theory Approach. Collectively, we propose these factors constitute the 'external reality' in relation to self-management behaviour.

Page 5 – 6 (Results Section)

The meticulous process of systematically sifting through large amounts of data led to the creation of multiple layers that define the distinct concept of external reality (Table 3). Furthermore, a stepwise agglomeration of codes into subcategories, followed by the deductive creation of unique themes, appeared to add more depth to external factors that impact self-management behaviour in individual with T2D. The iterative examination of narratives from FGDs (patients) and IDIs (healthcare providers) based on the similarity of context led to a more nuanced explanation to each subcategories that was consistent with dominant trends (vis- à-vis the experience of medication utilization and patient-doctor relationship) linked to self-management practices in literature[18, 19].

*(continued next page)*

Figure 1 describes a theoretical framework for understanding how individuals with T2D determine overall self-management behaviour. The interaction between the external reality (physical environment) and internal reality (inner psychological environment) and mediators of behaviour can be best described as a dynamic cycle that continue to shape and influence self-management behaviour (Figure 1). The results of this study are summarized in the upper half of this diagrammatic representation and consist of elements closely associated with the external realities of persons with T2D.

Within the context of external realities, three critical themes were identified, namely intrinsic background status, (ii) personal experience, and (iii) world view (Table 3). We developed the concept of intrinsic background status by merging codes that reflect societal, cultural, and communal factors that shape the daily living experience of people with T2D. On the other hand, the theme called personal experience was created by taking into account various dimensions that portray both physical and emotional experiences that were relevant to the phenomenon of living with T2D. Lastly, we define world-view as the personal perception of people with T2D about themselves and their perceived opinion about what society thinks about T2D. We will discuss the details of these subcategories in the subsequent sections of this article.

10. This seems inconsistent. Medication taking behaviors are modifiable. Availability or affordability of meds may not be modifiable (and its not therefore appropriate to call that adherence). Timing of medications can be modifiable. Health behaviors (often referred to as “lifestyle” in general are modifiable – that’s the whole point of diabetes care and education. So “fixed background status” and “lifestyle” don’t seem to fit together.

I would like to thank the reviewer for this insightful comment. We agree that there is a mix of both modifiable and non-modifiable factors in this particular theme. Hence, we amended “fixed background status” to intrinsic background status. Moreover, we have provided a broader operational definition in Table 3, we quote, “the social, cultural and community environment that shape the daily living experience of people with T2D”. We believe this definition/modifications encompasses both modifiable and non-modifiable elements seen in these subscategories.

11. This seems like something they are forgetting, rather than something that is fixed. With education, reminders, etc. it could be modifiable.

We have responded to this comment in Comment number 11

12. So age may be correlated with motivation to manage diabetes, yet is it a fixed relationship? I wonder if this section/theme needs to be worded differently to get your point across.

We have responded to this comment in Comment number 11

13. Are you referring to participants here, or people in general? Clarify and reword.

Thanks for pointing this out. We have revised our manuscript accordingly:

Page 11, Paragraph 3, Line 12 - 13

Most individuals with T2D (corroborated by information in interviews with pharmacists competent in providing T2D self-management services)

14. This doesn’t seem like the right word – it means giving in. Do you mean “agree” here?

Thanks for this comment. We have revised this sentence accordingly.

15. This quote does not support the last sentence in the previous paragraph. I would split that previous paragraph up, put the “They also reckon that…” sentence after the quote and edit it to something like “On the other hand, they also reckon that…”

We agree with this comment. We have made the necessary revisions to the manuscript (we have added an additional quotation to substantiate narrative in our result section):

Page 12, Paragraph 3, Line 14 - 25

Similarly, people with T2D feel the level of education is not a measure of eagerness to control their disease.

“Look, even if you hold a master’s degree, there is no guarantee you will look after your own wellbeing. I think there is no relationship between being a responsible patient and owning a degree. No I don’t think so” (IDM 015)

However, people with T2D do agree that a lower education background could likely lead them to rely more on their doctor's advice. On the other hand, they also reckon that more educated patients are well placed to make their own decisions about their disease management.

“These individuals (if they are highly educated) believe they have enough knowledge. As a result, they become extremely confident. So, they tend to do things their own way. For example, if they consider something is good for their health, they will generally follow through on their decision to comply” (IDM 021)

16. Define what you mean by this (judgmental label) and use that instead. Perhaps “…some participants with elevated A1C…”. How do you know it’s deliberate? If this is not part of the direct quote, please drop the word for methodological rigor/integrity. If “deliberately” was part of the quote, remove the brackets.

Thanks for this comment. We have edited the manuscript to avoid these terms:

Page 14, Paragraph 4, Line 20-24:

In a similar vein, some individuals with T2D with elevated HbA1c levels have a higher degree of sensitivity to the specific physical manifestation (lethargy, frequent micturation, hypoglycaemia or body aches) while some patients remain completely oblivious. These symptoms appear to be more prevalent when they “keep missing medications for a prolonged period of time”.

17. Might want to explain (briefly) what “modern” medications are…

Thank you for pointing this out. We have replace “modern” with the word “allopathic” in the text.

18. I’m not sure what the “exemplar” part of this refers to…

- Thank you for your comment. We have revised this section to provide more clarity to what was being discussed.

Page 14, Paragraph 4, Line 23-36:

The subcategory exemplar represents a person or life event from which a specific behavioural pattern is emulated from. In this instance, we discovered that people with T2D use cues derived from peer interactions or life experiences to inform their beliefs surrounding self-management practices.

19. Unless these nurses have diabetes, this is incorrect and inappropriate. Even if they do have diabetes, we are working hard to remove the word “diabetic” (see language use paper referenced above) and would no longer refer to them this way. You can simply say “nurses” here.

Thanks for this comment. We have omitted the word “diabetic” from the text.

20. You could just choose two of these as they all address the same points/theme.

- We agree with the reviewer. We have removed 2 quotations from the manuscript.

21. Missing narrative on services

- This details of this subcategory was described under the subheading “opinion about primary care services”. We moved this section and placed it beneath the “relationship with GP” subheading to reflect consistency with the order specified in Table 3.

22. Could use one or the other of these two quotes.

- As suggested, one quotation has been omitted from the text.

23. What is the conventional patient?

We have extensive edited the entire paragraph to provide a more accurate summary of the results in this study.

Page 20, Paragraph 1, Line 7-15:

The results from this study is an extension of a larger qualitative inquiry designed to decipher the intricate web of interaction behind the dynamics of self-management practices in T2D patients[9, 10]. In this article, we demonstrate that self-management behaviour in individuals with T2D consists of a rich interplay between factors associated with their external reality. (Figure 1)[9, 10]. Thus, this framework helps 1) summarize this behavioural complexity within an operational platform and 2) demarcate clear margins amenable to behavioural intervention or preventive measures.

24. Have to be careful with statements like this, as qualitative study results cannot be generalized. Reword to indicate this is what you found in this group of people in one study.

We agree with this comment. We have made revisions to the manuscript as illustrated below:

Page 20, Paragraph 1, Line 14-15:

More importantly, we summarized the thoughts and beliefs in relation to the external realities that shape the daily lives of a group of people with T2D.

25. This is not the same list as above… This seems to be the frist time you’ve differentiated between modifiable and non-modifiable. I had to go back up to the table and now I’m confused.

- We apologize for this confusion. In our response to Comment 11 above, we have made an attempt at revising the operational definition of intrinsic background status in Table 3 to reflect better inclusivity. Upon analysis of the results of the study, we found that it was appropriate to conclude these findings in the discussion section (i.e. that there is a clear distinction between the these subcategories). Hence, behaviour that are potentially amenable to intervention is labeled “modifiable” while others that require larger structural changes/reform are seen as “non-modifiable”. The response to Comment 27 below elaborates further on how this differentiation is crucial to clinical practice and effective policy changes.

26. Need more discussion. Tie this all together. It’s a LOT of information (findings) and yet how can I use this in practice?

- Thank you for this insightful comment. We have summarized how both clinicians and public health officials can benefit from this information in one paragraph:

The findings in our study have several implications for clinical practice and health policy planning. In the search for real-world solutions to challenges that arise from their external reality, we analyzed and condensed the complexity of diabetes self-management into actionable health outcomes by using a problem-focused approach. These clinical measures form a part of a logic model that outlines causal relationships among variables related to their external reality[6]. For clinicians, these measures provide tools for targeted customization for an opportunistic consultation aimed at sustained behavioural change. On the other hand, public health officials could use this information as a source for intervention mapping by setting goals that consist of a balance betweem modifiable factors (amenable to conventional health promotion strategies) and non-modifiable (possibly requiring a long-term structural reform and legislative process)[6]. Thus, our framework helps 1) summarize this behavioural complexity within an operational platform and 2) demarcate clear margins amenable to behavioural intervention or preventive measures.

27. Define what you mean by “optimal control” and reword

We have replaced this phrase with “effective T2D control”.

28. This sounds incredibly judgmental. How could you say this in a more empowering way?

We have phrased this sentence in a more empowering manner:

Page 21, Paragraph 3, Line 29:

To this end, GPs play an important role in 1)helping patients overcome challenges to diabetes self-management and 2) ensuring a mutually beneficial provider-patient interaction that facilitate behavioural change.

29. Yet it does not limit the ability to be supportive and encouraging. There is research showing this is not true. Could reword that there is a perception that this is true. What happens in time-constrained visits is that health professionals tend to revert to a disease-centered approach, rather than a person-centered approach. This is not absolute and does not have to happen.

We agree with the reviewer. We have edited the manuscript to reflect these changes:

Page 22, Paragraph 2, Line 5-14:

Our study highlights the paternalistic doctor-patient communication styleprevalent in most Southeast Asian countries[21]. Similarly, health services are overwhelmed by high patient load which severely limits constructive consultation time[21]. This situation in part gives rise to patients’ account of grievances with GPs described in the form of 1) having received insufficient advice, 2) limited consultation time and 2) GPs reportedly being poor mannered or inattentive[22]. However, it is equally important to note that a time-constrained clinical environment does not entirely preclude a more participatory role of consultation. Moreover, high quality patient-provider communication (characterized by non-judgemental acceptance, open and honest interaction) has shown to bring about better self-management behaviour in individuals with T2D[22]. .

30. This sounds like someone is breaking the law. This language perpetuates all the problems you are reporting here. People with diabetes are simply making choices, not “complying” or “adhering.” Because they are treated like criminals for eating something that raises their glucose, they feel discouraged, chastised, sad, angry, frustrated and often want to (and do) give up. We have to change these messages.

- This is an insightful comment. We have edited the sentence accordingly:

Page 22, Paragraph 3, Line 19 - 21:

This phenomenon is evidenced by not complying with dietary adherence despite being fully aware of the consequences of their actions .

31. I don’t know what this sentence means (Strikingly, an offshoot of this larger study discovered a close association between both food addiction scores and binge eating severity with the diabetes status of the patient

We have edited this sentence to provide more clarity:

Page 22, Paragraph 3, Line 22 - 24

These behavioural patterns suggest a close parallel ro pioneering work from the field of food addiction[23]. close association between both food addiction scores and binge eating severity among persons with T2D[24].

32. Persuasion does not equate to empathy. In fact it can be the opposite (coercion). What about approaches like motivation interviewing? Strengths-based and person-centered approaches? Empowerment? Lots of research on these. This is a label and would make other people feel like failures.

We thank the reviewer for this comment. We have extensively revise this section by following the suggestions as indicated by the reviewer:

Page 23, Paragraph 1, Line 3 - 13

One plausible would be the use of context-specific (by promoting the importance of weight control or physical activities) motivational interviewing techniques to produce a more meaningful and empathetic experience for both GPs and individuals with T2D[25]. This intervention is likely to improve diabetes-related outcomes when integrated with person-centered self-management education. Notably, these positive effects can be further enhanced by addressing negative perceptions and adverse disease experience via group based self-management education with popular opinion leaders (i.e. T2D individuals with good control and expert physicians) using interpretations from real-world parameters depicted in this study[26, 27].

*Comments from Reviewer 2*

1. A qualitative grounded theory approach“ ist double formulated. I don’t know a quantitative GT.

- We would like to thank the reviewer for pointing this out to us. We have replaced this terminology accordingly in the introductory section

Page 3, Paragraph 3, Line 22:

In this study, we aim to develop a **qualitative** theoretical model that could explain disease management in T2D patients[9]

2. Purposive sampling is not the sampling approach for Grounded Theory. This is the theoretical sampling. This deviation should be well justified and should be mentioned in the limitations in my opinion.

- We thank the reviewer for this comment. Allow us to clarify. Qualitative inquiry using a Grounded Theory approach is a very dynamic process. In line with the process we employed a stepwise strategy
- We first employed a purposeful sampling to “select participants because they are information-rich and illuminative, that is, *“they offer useful manifestations of the phenomenon of interest“* (Quinn – Qualitative Research & Evaluation Methods, 4^th^ Edition, pg 46).
- In accordance with Grounded theory approach, we coded our data from the very outset. Ultimately, we have identified a plateau in emerging theories after the 10^th^ participant (patient). And only then, we performed theoretical sampling to (we quote Charmaz) *“develop your emerging theory, to elaborate and refine categories constituting your theory”* (Charmaz – Constructing Grounded Theory, pg 96)
- Hence, what we employed in this study was a common stepwise strategy in Grounded Theory, which would also answer doubts about our Topic Guide (which was revised at least 13 times within the first 10 interviews prior to theoretical saturation, discussed in a separate comment).

3. Among the participants, it is not quite clear to me why doctors, diabetes pharmacists and pharmacists were questioned.

- We thank the reviewer for this comment. Interviews of health providers who we were trained (GPs, pharmacists and nurses) to provide care for persons with Type 2 diabetes were deliberate. We have explained this in detail in the subsection labeled “trustworthiness” in our manuscript. In summary, we sought to improve the validity of our qualitative data through a series of data triangulation (not limited to only in-depth interviews with healthcare providers) to verify the information that we obtained from individuals with T2D.
- We appended the passage from our manuscript below:

Page 7, Paragraph 3, Line 20 - 26:

Trustworthiness of this study was assured by using different techniques of data collection through both in-depth interviews and FGDs (methodological triangulation)[12]. Two researchers performed data analysis and interpretation (investigator triangulation). We chose to validate the information (data triangulation and negative case analysis) by comparing interviews that were obtained from diverse sources (patients and healthcare providers). Additionally, the authors interpreted, conceptualized and revised behavioural constructs at various stages of the study via an iterative process (persistent observation).

4. When it comes to self-managment beliefs in T2D patients, the external perspective irritates me. If it needs it, please explain it better. I do not understand it and find it highly problematic. Were the different sources brought together? The title of the paper raises doubts about the usefulness of the expert information.

- We apologize for the lack of clarity in the title of our paper. We have edited the introductory section to help clarify what it meant by 'external reality'.

Page 3, Paragraph 3, Line 22 - 29:

This study builds upon a larger initiative aimed at developing a comprehensive qualitative theoretical model that could help explain disease management in people with T2D[9, 10]. In previous papers, we have expounded on the drivers of 'internal realities' related to self-management behaviour namely, 1) specific mediators, and 2) observed performance self-care activities[9, 10]. In the current article, we intend to expound on the immediate physical and sociocultural that dictate the naturalistic environment of T2D patients through a Grounded Theory Approach. Collectively, we propose these factors constitute the 'external reality' in relation to self-management behaviour.

5. Why were the same patients questioned in focus groups in addition to the individual interviews? What were the topics?

- Thank you for this comment. The purpose for this step (as mentioned earlier in relation to “trustworthiness”) was to verify and consolidate the validity (via focus group discussions) of emergent theories that we obtained throughout our in-depth interviews with persons with T2D. This process of “verification” forms a part of a strategy called “methodological triangulation”.
- The topic guide for this focus group discussion is found in Supplemental File 1

6. And so it leads straight over to my biggest question or my biggest concern: What did you actually ask in the interviews? You write they are in-depth interviews. That is where a narrative-generating impulse is set. Which one is that? I can't find it in the manuscript. Can you give us your central question. A little later I read "topic-guide". This does not fit to in-depth interviews. That would be semi-structured interviews. Following your advice, I looked at the study protocol and the topic guide. Afterwards I would very much question the term in-depth interview.

- Thank you for your comments. We apologize for this ambiguity. Allow us to explain. The topic guide described in the protocol paper is a but a preliminary “prototype”
- Initially, we used the initial topic guide furnished along with the manuscript. However, we have revised this topic guide to reflect changes that were seen during the in-depth interviews based upon the first topic guide. Gradually (and in accordance with emergent nature of a Grounded Theory approach), we incorporated questions that we related to the emergent theories found in our interviews. In summary, we performed approximately 13 revisions to the topic guide. Finally, we utilized this final version of the topic guide during the theoretical sampling phase.
- To explain what is meant by our “central question”, our reply to this is as follows – it was dependent on the evolution of our data throughout the qualitative inquiry. As the reviewer rightly pointed out, we began by exploring only aspects related to (and not limited to) medication compliance. However (as expected via the iterative analytical processes of a Grounded Theory approach), we dynamically improvised the line of question (which was constantly informed by emergent data). Hence, we constantly revising the content of our topic guide until the initial point of data saturation.
- In retrospect, we found ourselves exploring the larger theme of self-management beliefs as opposed to medication compliance as suggested by our protocol paper. Having said that, the processes outlined in the protocol paper remained the same throughout our study.

7. Furthermore, you write that you had a theoretical saturation after 10 interviews, but then you explored and enriched this with 14 more interviews. Is it perhaps because of the sampling that so many more interviews had to be conducted?

- Thanks for your comments. After 10 interviews (purposeful sampling), we reached theoretical saturation. However, we needed to build on the existing theme uncovered during the first 10 interviews. Thus, we entered the stage of theoretical sampling in a bid to *“develop our emerging theory, to elaborate and refine categories constituting your theory”* (Charmaz – Constructing Grounded Theory, pg 96). A broader explanation to this comment can be found in our response to Comment number 2 above.

8. The description of the analysis does not go beyond the formation of categories. How does the theory emerge?

- Thanks for the comment. This detailed process was already included as Appendix B and C in the previous submission. We include all these files as Supporting Information in the current submission (refer to S1 Table, S2 Table and Topic Guide). The stages coding that led to the generation/formation of categories is consistent with the procedures outlined (and referenced in our manuscript) extensively in our protocol paper (i.e. open coding, axial coding and selective coding). We followed standard Grounded Theory techniques (Charmaz – Constructing Grounded Theory, pg 43 - 71) to categorize and form codes. The structured formation of categories was already highlighted in the previously included Appendix 2 where we demonstrate how quotations are transformed into themes (sub-categories) and finally into 3 core phenomena (categories).

9. I don‘ understand how do you observe „thought patterns“.

- Thanks for this comment. We have made changes to the sentence accordingly.

Page 8, Paragraph 1, Line 7 - 8:

Furthermore, the narratives from FGDs (patients) and IDIs (healthcare providers) helped consolidate the linkages between various themes (Table 3) on the basis of similarity of context.

10. Table 3: Why is lifestyle one of the non-modifiable factors?

- Thank you for this comment. This error was an oversight on our part as exemplified discussion section (Pg 17, Paragraph 1, Line 7) where we have already classified lifestyle as a modifiable factor in the original version of the manuscript. In addition, we have edited Table 3 accordingly.

Table 3:

**Intrinsic background status**

Social, cultural and community environment that shape the daily lives of the patient

11. Chapter lifestyle: „In general, patient frequently miss taking their medikation when they

are out of their homes. You cannot introduce such a statement with "in general". What do you base that on, 24 interviews? With this statement, you are overstressing your data.

- Thanks for this comment. It was not our intention to overstate the findings of our data; the phrase “in general” in the sentence was written in relation to the participants within our study and certainly not an interpretation of what is seen in the general population. We have corrected this error accordingly.

Page 11, Paragraph 3, Line 14:

Individuals with T2D in our study commonly experience very little side effects with their medications.

Page 9, Paragraph 1, Line 4:

In our study, persons with T2D frequently miss taking their medication when they are out of their homes.

Page 11. Paragraph 3, Line 14

Individuals with T2D in our study commonly experience very little side effects with their medications.

12. The presentation of the results seems very descriptive, not very interpretative. So not so much based on Grounded Theory. I miss the merging of the areas; categories are not developed in further details. I cannot reconstruct how Food environment, lifestyle and sociodemographics became the background.

- Thank you for pointing this out. Described below, we have written a preamble to the detailed description of the results section which highlights the deductive/intuitive processes involved in creating major themes and subthemes.
- Additionally, we have provided an operational definition to most subcategories and how they were developed in the results section.

Page 11, Paragraph 3, Line 14:

The meticulous process of systematically sifting through large amounts of data led to the creation of multiple layers that define the distinct concept of external reality in our study (Table 3). Furthermore, a stepwise agglomeration of codes into subcategories, followed by the deductive creation of unique themes, appeared to add more depth to external factors that impact self-management behaviour in individual with T2D. The iterative examination of narratives from FGDs (patients) and IDIs (healthcare providers) based on the similarity of context led to a more nuanced explanation to each subcategories that was consistent with dominant trends (vis- à-vis the experience of medication utilization and patient-doctor relationship) linked to self-management practices in literature[15,16].

Figure 1 describes a theoretical framework for understanding how individuals with T2D determine overall self-management behaviour. The interaction between the external reality (physical environment) and internal reality (inner psychological environment) and mediators of behaviour can be best described as a dynamic cycle that continue to shape and influence self-management behaviour (Figure 1). The results of this study are summarized in the upper half of this diagrammatic representation and consist of elements closely associated with the external realities of persons with T2D.

*(continued on nest page)*

Within the context of external realities, three critical themes were identified, namely intrinsic background status, (ii) personal experience, and (iii) world view (Table 3). We developed the concept of intrinsic background status by merging codes that reflect societal, cultural, and communal factors that shape the daily living experience of people with T2D. On the other hand, the theme called personal experience was created by taking into account various dimensions that portray both physical and emotional experiences that were relevant to the phenomenon of living with diabetes. Lastly, world-view is defined as the personal perception of people with T2D about themselves and their perceived opinion about what society thinks about T2D. We will discuss the details of these subcategories in the subsequent sections of this article.

13. I cannot understand the first sentence at all. Where does it come from, I did not read it out at all. I also can't find the content of the second sentence (or the background for it) in the description of the result at all.

- Thank you for this comment. We have edited the entire paragraph and added citations that might provide more clarity to the reader. The correction made is specified below:

Page 18, Paragraph 2, Line 10 - 16

The results from this study is an extension of a larger qualitative inquiry designed to decipher theintricate web of interaction behind the dynamics of self-management practices in T2D patients[9, 10]. In this article, we demonstrate that self-management behaviour in individuals with T2D consists of a rich interplay between factors associated with their external reality. (Figure 1)[9, 10]. Thus, this framework helps 1) summarize this behavioural complexity within an operational platform and 2) demarcate clear margins amenable to behavioural intervention or preventive measures.

14. The interview guide, with its strong focus on medication, does not seem to me to be so well aligned with the cognitive interest of the overall project. How the answers to these questions are to be used to develop a theoretical model that explains disease management in diabetics is questionable.

- Please refer to our responses to Comments 5,6 and 7

15. I come back to the introduction. Strictly speaking, it already seems to fit to manuscript. But I as a reader remain somewhat confused with my question: what is the overall question of the project, what is the question of the manuscript, how can the result presented in the manuscript contribute to the overall question.

- Thank you for this comment. We have edited the entire paragraph and added citations that might provide more clarity to the reader. The correction made is specified below:

Page 3, Paragraph 3, Line 25 - 29

This study builds upon a larger initiative aimed at developing a comprehensive qualitative theoretical model that could help explain disease management in people with T2D[9, 10]. In previous papers, we have expounded on the drivers of 'internal realities' related to self-management behaviour[9, 10]. This analysis led to the generation of 2 main categories – 1) internal reality (the inner psychological environment and personal volition), and 2) mediators of behaviour (Figure 1). In the current article, we turn our attention to the exploration of the immediate physical and sociocultural environment of individuals with T2D and their influences on self-management behaviour.
