# Supplementary material for: The external realities of people with type 2 diabetes—Understanding disease perspective and self-management behaviour via Grounded Theory Approach
Source: PLoS One. 2021 Jan 14;16(1):e0245041. doi: 10.1371/journal.pone.0245041 (PMC7808602; doi:10.1371/journal.pone.0245041)
Supplement: Attachment — Submitted filename: Response To Reviewers (16.12.2020).docx [file pone.0245041.s008.docx]

**Response to Reviewers**

*Comments from Reviewer 2*

1. I take a very critical view of the topic guide for the interviews with their strong focus on medication.

- We thank the reviewer for pointing out this crucial element of our study to us. We have offered an explanation below (with a flowchart at the end of this document) to help clarify what we performed in our study. Additionally, we have highlighted several sections of the topic guide (please refer to the updated Supporting Information S1 file) to provide more detail about what was done during the course of our research.
- From the *a priori* literature review we performed, we identified that medication adherence forms approximately 70 – 99% of self-management activities (Coyle et al., 2013; Mosnier-Pudar et al., 2009). Therefore, we attempted to carefully craft the topic guide to reflect what is seen in reality, emphasizing medication adherence.
- Having said that, this statement does not imply that we have sidestepped the need to explore other related paradigms as well (e.g., diet/food environment, self-monitoring of blood glucose, belief system and the salience of physical activity etc.). We have explored these themes, evidenced by what is seen in our topic guide for both in-depth interviews (IDI) and focus group discussions (FGD). Please refer to the updated Supporting Information S1 – Topic Guide.
- The interview process can be seen as a checklist phase (initial questions), followed by a theoretical sampling process (questions from emergent themes). The FGDs serve to confirm, confirm, and derive a consensus to what was found in the IDIs. Moreover, the topic guide used in FGDs (refer to highlighted aspects of the topic guide) further substantiates our exploration of other equally essential self-management activities. To clarify and explain this process in detail, we have incorporated a flowchart into our manuscript.

2. Regarding my problem with the inclusion of health care professionals, I was referred to a chapter in the manuscript. However, it remains open for me what external parties can contribute to the questions of the study and what questions were asked to the experts in their individual interviews. None of this clarified the revision for me.

- We thank the reviewer for this comment. Please allow us to clarify the role of healthcare professionals (HCP) in this study with the flowchart below.
- From the illustration in Figure 1 below, it is evident that interviews HCPs were meant to verify or corroborate what was being narrated by individuals with diabetes (i.e. main process of the study). This step forms the foundation of methodological triangulation (Triangulation 1) where HCPs (third party observers) provide their input on specific characteristics of the patient based upon a similar topic guide (but tailored to HCPs) designed from what was collected in Step 1. Therefore, this process reduces the speculative nature of our interviews and provides better validity to the narrative provided people with diabetes.

3. Somewhere in the "strenghs and limitations" I am reading that the statements of people with diabetes were submitted to the experts for verification. Why should this happen when it comes to the "realities" of people with diabetes.

- Thank you for this comment. We would like to clarify what was meant by “submitted to experts for verification below (included in the manuscript as Supporting File 2)
- As depicted in Figure 1 below (Triangulation 2), once we have established the core phenomenon and themes to our study as described by individuals with Type 2 Diabetes in our interviews, we sought to establish the trustworthiness of through methodological triangulation. Hence, we created a codebook so that experts (in the field of diabetes self-care) would further examine and provide feedback to the authenticity of our findings (inter-rater reliability). These experts comprise academic editors who were tasked at cross-checking the consistency of information derived from qualitative inquiry. These auditors also checked whether the analysis was in line with accepted standards and examined the analysis process and records for accuracy.
- We have also revised the specific sentence in the manuscript to provide more clarity:

*Page 8, Paragraph 1*

Also, themes derived from this study were assessed by a panel of experts to establish coding uniformity and was found to have excellent content consistency (Cohen's κ =0.88).

4. In the results section I miss the framing. Incredibly many small individual dimensions are opened and quotes are added.

- We thank the reviewer for pointing this out. We have added more quotations to the manuscript to provide more clarity the dimensions we have described in the manuscript. Moreover, we have also included additonal quotations for each dimensions in Supporting Document S4.

*Page 10, Paragraph 2*

“I am a police officer. In a day, there are just far too many activities I am involved in. At one point I am always at the office, and before you know it I am out doing field work. So sometimes I miss taking my medications due to that” (IDM 023)

*Page 11, Paragraph 4*

“A person with little education will look upon the advice of the doctor as the words of the Gospel. They will follow the doctor’s advice in a very rigid manner. Just like my mom” (IDM 012)

*Page 12, Paragraph 4*

“When we consume meals from food outlets or at the cafeteria while working away from home, we don’t really get to choose proper food doctor. So, the reading [blood glucose] will increase and then decrease sometimes” (IDM002)

*Page 13, Paragraph 1*

“I can still consume this medication, but for me, I feel it makes me feel lethargic and tired” (IDM 019)

*Page 14, Paragraph 2*

“Right now, it’s under control [diabetes]. But I just feel it can go up [blood glucose level] at any time” (IDM 002)

***Emergent themes from In-Depth Interviews (IDI)***

A review of scientific literature was conducted to identify areas that define self-management activities

A preliminary topic guide was designed and emergent themes were elicited via Ground Theory Approach

After the 10^th^ interview, no additional themes were discovered and theoretical saturation was considered to have been reached

The topic guide was revised

***Triangulation 1***

***Concurrent revision of topic guide***

***Methodological Triangulation***

A preliminary topic guide was designed and emergent themes were elicited via Ground Theory Approach from individuals with diabetes

After the 10^th^ interview with healthcare providers, no additional themes were discovered and theoretical saturation was considered to have been reached **(negative case analysis)**

***10^th^ iteration of topic guide***

***Theoretical Sampling (IDI)***

Additional interviews with individuals with diabetes

14 interviews

Emergent themes explored, enriched and consolidated

Development of topic guide for focus group discussions

***Conceptual Framework***

***12^th^ iteration of topic guide***

***Data Triangulation***

Transcribed interviews coded by researcher

Cross discussion with collaborators to establish themes **(persistent observation)**

Inter-coder reliability testing **(investigator triangulation)**

Framing of themes

Codebook generated

Kappa inter-rater reliability/expert consensus

***Focus Group Discussions (FGD)***

Total of 12 individuals with diabetes in 2 sessions

All themes derived from IDIs tested and verified

***Triangulation 2***

**Figure 1: Flow chart of the study design and development of conceptual framework**
